# Immersive futures in healthcare: A mapping review of review articles on the metaverse

**DOI:** 10.1177/20552076261431602

**Published:** 2026-03-13

**Authors:** Pauliina Rikala, Minna Ylönen, Mads Solberg, Charlott Sellberg, Ville Heilala, Teuvo Antikainen, Miguel Munoz, Tommi Kärkkäinen, Raija Hämäläinen

**Affiliations:** 1 Department of Education, 4168 University of Jyväskylä, Jyväskylä, Finland; 2 4169 Hospital Nova, Wellbeing Services County of Central Finland, Jyväskylä, Finland; 3 Department of Health Sciences, 8018 Norwegian University of Science and Technology, Ålesund, Norway; 4 Department of Applied Information Technology, 3570 University of Gothenburg, Göteborg, Sweden; 5 Department of Social Sciences and Philosophy, 4168 University of Jyväskylä, Jyväskylä, Finland; 6 Faculty of Information Technology, 4168 University of Jyväskylä, Jyväskylä, Finland

**Keywords:** Metaverse, immersive technologies, intelligent technologies, healthcare, mapping review

## Abstract

**Background:**

The metaverse has the potential to transform healthcare and healthcare education by offering immersive, interactive experiences. As research on the metaverse rapidly expands, a synthesis is required to understand its current state, trends, and future directions in healthcare.

**Methods:**

We conducted a mapping review of existing review studies on the metaverse in healthcare using topic modeling, hierarchical clustering, and qualitative interpretation. This was complemented by computational text analysis to examine thematic developments and structural patterns.

**Results:**

The analysis yielded three distinct clusters: (1) immersive therapeutic and educational applications with intelligent integration, (2) immersive technologies for surgical training and clinical simulation, and (3) integrated, immersive, and intelligent technologies for personalized, networked healthcare. These clusters illustrate a shift from conceptual exploration toward applied, system-level integration. Applications show promise in mental health, surgical education, and personalized care, among others, but the evidence is preliminary. Key risks include privacy concerns, governance gaps, and equity challenges.

**Conclusions:**

As enthusiasm for metaverse technologies grows, it is crucial to ensure that optimism does not outpace evidence and readiness. The metaverse offers significant opportunities for human-centered healthcare and professional training, but it requires rigorous validation, ethical frameworks, and inclusive design. To ensure responsible adoption and a sustainable impact, it is critical to align developments with the WHO's strategic objectives of collaboration, implementation, governance, and human-centered systems.

## Introduction

Digital technologies are evolving rapidly, influencing nearly every aspect of daily life and reshaping our understanding of the world.^1^ In healthcare, technological innovations have transformed how professionals diagnose, treat, and manage care,^2,3^ making services more efficient, accessible, and equitable.^4^ A promising step in technological development is the metaverse. Although this concept was first introduced in Stephenson's fictional novel *Snow Crash* (1992),^5^ the metaverse has since become a tangible digital environment that blurs the line between reality and science fiction.^6,7^ As the term suggests, it is a combination of *meta* (Greek prefix meaning beyond) and *universe*, referring to a post-reality digital environment that merges physical and virtual worlds.^8^ The metaverse represents an integrated ecosystem combining immersive technologies—such as virtual reality (VR), augmented reality (AR), mixed reality (MR), holography, and haptic devices—and intelligent systems, including artificial intelligence (AI), machine learning (ML), the Internet of Things (IoT), digital twins (DTs), wearable biosensors, cloud computing, and robotics. The aim is to create rich, interactive, multisensory experiences^9–13^ that are personalized and engaging^14^ and stimulate sensory experiences such as sight, sound, touch, temperature, and balance.^11^

Thus, the metaverse exists within a broader conceptual landscape that includes immersive technologies, intelligent systems, and digital healthcare frameworks. Although these concepts influence metaverse development, they are not synonymous. Immersive technologies have been widely studied for their applications in pain management, stroke rehabilitation, mental health, cancer care, and neurodegenerative disorders.^15^ Furthermore, these technologies support visualization, treatment planning, and rehabilitation, thereby improving accessibility and offering therapeutic and preventive benefits.^15–18^ A growing body of evidence indicates that immersive technologies have beneficial effects across multiple domains, including pain reduction, improved functioning and mobility, enhanced psychological and neuropsychological outcomes, better quality of life, and altered physical sensations.^19^ They also enhance medical training, surgical planning, and anatomical exploration by offering safe simulation environments and real-time assistance during procedures.^16,20–26^ Studies have demonstrated that training based on immersive technology improves post-intervention knowledge and skills outcomes among healthcare professionals compared with traditional education or alternative digital modalities.^27^

Beyond immersive experiences, the metaverse integrates intelligent systems that enhance clinical insights and efficiency. Intelligent technologies enable personalized healthcare, predictive interventions, remote monitoring, and advancements in medical research.^28–33^ These systems replicate physical assets and processes,^26^ support adaptive therapies,^30–33^ and, in some cases, outperform human experts—for example, in tumor detection and clinical trial design.^34^ Meta-analytic evidence suggests that intelligent technologies can meaningfully reduce depression and distress, especially when delivered through multimodal, generative, and mobile-integrated systems. However, their impact on broader psychological well-being remains limited.^35^ Thus, while intelligent technologies have immense potential to revolutionize healthcare through personalized medicine, operational efficiency, and accelerated medical research, many applications remain at the proof-of-concept stage. This underscores the need for further research, data standardization, and validation efforts.^36^

Furthermore, despite advances, there remain ethical, technical, and societal challenges to be addressed.^10,37^ The integration of metaverse technologies into real-world healthcare systems is still in its infancy.^9^ Moreover, some scholars have questioned whether the metaverse represents a genuine paradigm shift or is primarily a marketing construct, noting its conceptual ambiguity and socioeconomic dimensions.^7–10^ While related concepts—such as healthcare 4.0,^38,39^ smart healthcare,^40^ u-health,^41^ and cyber-physical systems^42^—exist, our focus is on the metaverse because it represents a broader, integrated ecosystem that unifies immersive and intelligent technologies into an interactive, multidimensional environment.

**Figure 1.**
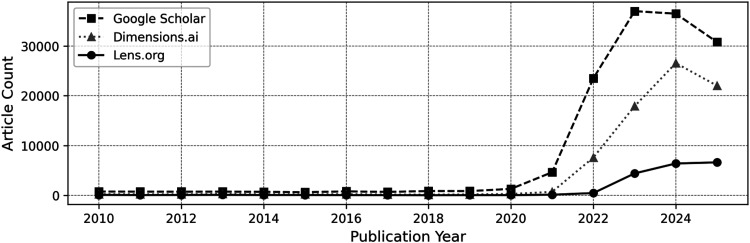
The annual number of publications on dimensions.ai, Google Scholar, and Lens.org identified with the search term “metaverse.”

Although the metaverse has not yet been identified as a central component of digital health by the WHO,^43^ interest in it is growing. A substantial number of review articles on the metaverse have been published;^13,44–50^ however, considerable variations exist in terms of scope, focus, and methodological rigor, thereby limiting the overall coherence of existing knowledge. Furthermore, although the existing literature often presents an optimistic view of the metaverse's potential, there is a lack of critical engagement with its limitations. To address this gap, we conducted a mapping review of English-language review articles examining metaverse applications in healthcare. This mapping approach^51^ is particularly suitable for identifying key themes and providing a structured overview as research interest grows (see Figure 1).

Integrating computational and qualitative analyses, this study provides a visual and thematic synthesis of current research by identifying key trends, conceptual clusters, and emerging directions to inform future studies and innovations. Because it is a mapping review of review articles rather than primary studies, the identified clusters represent groupings of secondary literature, excluding individual interventions or clinical trials. The evidence synthesized herein reflects conceptual and aggregated perspectives from extant reviews rather than direct validation of immersive and intelligent technologies. Consequently, a “big picture” perspective is adopted^52^ focusing on a descriptive synthesis rather than an effectiveness assessment. To enhance policy relevance, the findings are aligned with the WHO's Global Strategy on Digital Health (2020–2025; SO = Strategic Objective),^43^ which emphasizes collaboration and knowledge sharing (SO1), strategy implementation (SO2), governance (SO3), and human-centered systems (SO4). Our research question is as follows:

- What are the characteristics, publication trends, thematic structures, and application domains of the review literature on the metaverse in healthcare, as revealed through bibliometric and cluster analysis?

## Methodology

This mapping review followed the EQUATOR principles^53^ for transparency and, where applicable, adhered to the PRISMA guidelines,^54^ including the PRISMA-ScR checklist^55^ (see Appendix 1). Mapping reviews generally do not require formal protocol registration, since their objective is to provide a descriptive overview of the evidence landscape rather than to evaluate intervention effects. Therefore, no protocol was registered for this review. However, we designed and reported the review process in accordance with recommendations for mapping reviews, particularly those outlined by Li et al.^56^ and Cambell et al.^52^ The objective of this review was to systematically identify and map review articles on metaverse applications in healthcare, with a focus on their characteristics, publication trends, thematic structures, and application domains.

### Eligibility criteria

We included peer-reviewed review articles (meta-analyses and systematic, scoping, narrative, integrative, and umbrella reviews) published in English that explicitly addressed the metaverse as a central concept in healthcare. Eligible articles focused on healthcare delivery or the professional development of healthcare professionals. The complete inclusion and exclusion criteria are outlined in Table 1. Although systematic reviews are usually prioritized due to their methodological rigor,^57^ we included other review formats to gain a broader understanding of the subject matter. This decision was informed by the evolving and multidimensional nature of metaverse research in healthcare (see Figure 1).

**Table 1.** Article inclusion and exclusion criteria.

Inclusion criteria	Exclusion criteria
Peer-reviewed review articles (including meta-analyses and systematic, literature, scoping, narrative, integrative, and umbrella reviews)	Articles not classified as review articles (e.g., editorials, opinion pieces, discussions, correspondences, commentary pieces, theses, and primary studies)
Articles published in English	Articles not written in English
Articles that explicitly discuss the metaverse as a central concept in the context of healthcare, offering more than superficial mentions and providing added value through conceptual clarity, synthesis of findings, or novel insights relevant to healthcare applications	Articles that mention the metaverse only in passing (e.g., in references or tangential contexts) without addressing it as a central concept
Articles that focus on either healthcare delivery—such as clinical applications, patient care, or therapeutic interventions—or healthcare professional development, including training, education, skills enhancement, or workplace learning	Articles that mention the metaverse but do not relate it to healthcare or professional development

### Information sources

Dimensions.ai, Google Scholar, and Lens.org were selected based on their user-friendly, flexible interfaces and extensive peer-reviewed coverage.^58,59^ Searches were conducted in two phases. The first phase, which took place in May 2025, employed the Dimensions.ai platform. However, the extent of its coverage was limited, with only 17% of the results overlapping with those of Google Scholar. Consequently, the subsequent phase, conducted in August 2025, expanded the search to include Google Scholar and Lens.org.

### Search strategy

The initial search strategy included four sets of keywords: (1) metaverse, (2) professional development, (3) review types, and (4) professional context. Regarding “metaverse,” the search was limited to this term because the goal was to examine how this concept has been integrated into various technologies in healthcare rather than to review related but distinct concepts, such as extended reality (XR). While there are numerous studies on these immersive technologies in this context, these concepts do not fully represent the metaverse as an integrated ecosystem. We focused specifically on publications that explicitly addressed the metaverse concept and its integration with multiple technologies rather than isolated immersive technologies. In the first phase, we applied a four-concept query with extensive synonyms in Dimensions.ai. When we tested the same query in Google Scholar, this yielded over 10,000 results, many of which were irrelevant (e.g., travel and library science). To improve precision, we refined the strategy in the second phase (August 2025). Adjustments, including simplifying keyword combinations, were necessary because search syntax and filtering options vary significantly across databases. The differences in database syntax and filtering options, combined with the complexity of the initial search strings, may have influenced the comprehensiveness of the retrieved publications. Despite applying refinements to improve precision, some relevant research may have been overlooked. This should be considered when interpreting the scope and completeness of the findings. The full search strings and dates for all databases are reported in Table 2.

**Table 2.** Search strings and parameters used with dimensions.ai, Google Scholar, and Lens.org.

**Database**	**Date**	**Search string**	**Filters**	**Results**
Dimensions.ai	May 14, 2025	(“professional development” OR “professional training” OR “professional learning” OR “workplace learning” OR “workplace training” OR “staff training” OR “career growth” OR “skill enhancement” OR “skills development” OR “staff development” OR “on-the-job training” OR “continuous learning” OR “professional competence”) AND (“metaverse”) AND (“review article” OR “literature review” OR “systematic review” OR “meta-analysis”) AND (“healthcare professionals” OR “health care professionals”)	English; Publication Type: Article; Document Type: Review Article	116
Google Scholar	Aug 17, 2025	"metaverse” AND (“review article” OR “systematic review”) AND (“healthcare professionals”)	None	3490
Lens.org	Aug 17, 2025	metaverse AND ((“review article” OR “systematic review”) AND (“healthcare professionals”))	Scholarly Works	77

### Selection process

The search yielded 3683 results: 116 with Dimensions.ai, 3490 with Google Scholar, and 77 with Lens.org. The Dimensions.ai search yielded only English-language review articles. Therefore, no initial filtering was applied, and all 116 records were retained for screening. Google Scholar and Lens.org retrieved a wider variety of source types, such as books, editorials, opinion pieces, correspondence, theses, and primary studies. Following the best-practice guidance of Haddaway et al.^60^ for Google Scholar, we screened only the first 200 results because relevance substantially decreases beyond approximately 200–300 hits, and additional screening is unlikely to identify more eligible studies. Accordingly, the Google Scholar results were restricted to 200 records for screening feasibility. For Google Scholar and Lens.org, we applied an initial filtering step to remove duplicates, non-English publications,^61^ and non-review items prior to screening. Clearly off-topic records (e.g., football or marketing) and one record without an abstract were also excluded. In total, 184 records were removed before screening.

In Phase 1 of screening, two authors (MY and PR) independently screened the studies, with each author reviewing 58 of the abstracts. After screening the first few abstracts, the authors cross-checked their decisions using a shared Excel spreadsheet. This spreadsheet tracked eligibility decisions and ensured transparency. In cases of uncertainty, the two authors assessed the articles collaboratively. After the title/abstract screening, 66 records remained. These records were sought for retrieval and assessed in full text using ATLAS.ti. During the full-text screening, we checked for the presence of the term “metaverse,” using the search functions in ATLAS.ti, which led to the exclusion of 32 papers that did not mention the term. PR screened 24 records and MY screened 10, with five assessed collaboratively. Nineteen studies met the inclusion criteria.

In Phase 2, one author (PR) screened 277 titles and abstracts using the same eligibility criteria and procedures as in Phase 1. The decision to use single screening was driven by feasibility and timelines. Evidence from methodological evaluations indicates that single screening performed by experienced reviewers can be an acceptable tradeoff for efficiency in rapid or resource-limited reviews. However, it may miss a small proportion of studies compared with double screening.^62^ Uncertain cases were discussed with MY to reach a consensus. After the initial filtering process, 93 records remained for title and abstract screening. Of these, 2 were inaccessible and 36 were excluded because they were irrelevant to the topic or focus. Fifty-five full texts were assessed; 2 had insufficient depth or scope, 13 were irrelevant, and 10 were not reviews. This resulted in 30 included studies.

**Figure 2.**
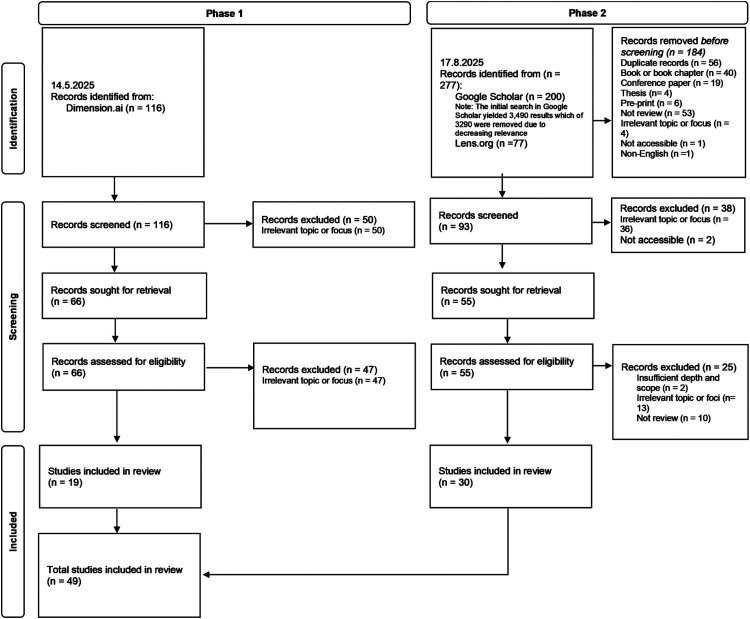
Flow diagram showing the review selection process.

Table 3 provides detailed reasons for exclusion at each stage. The PRISMA-style flow diagram (Figure 2) offers a summary of these reasons. Across both phases, the most common reason for exclusion was an irrelevant topic or focus. Of the 116 Dimensions.ai records, 49.1% were excluded because the term “metaverse” was not mentioned, 23.3% were out of scope, and 11.2% only briefly mentioned the term. Of the 93 records screened after the initial Google Scholar and Lens.org filtering, the most frequent reason for exclusion was an irrelevant topic or focus (52.7%). Additional exclusions were due to insufficient depth and scope (*n* = 2), not being a review (*n* = 10), and being inaccessible (*n* = 2). The selection process for the included reviews is described in detail in Appendix 2.

**Table 3.** Reasons for exclusion across different screening stages.

	Stage	Reason for exclusion	N
Phase 1	Removed before screening		-
	Title/abstract screening	**Irrelevant topic or focus**	**50**
		Metaverse not mentioned	25
		Out of scope	25
	Full-text screening	**Irrelevant topic or focus**	**47**
		Metaverse not mentioned	32
		Metaverse mentioned only in passing	13
		Out of scope	2
Phase 2	Removed before screening	**Records removed before screening**	**184**
		Duplicate records	56
		Book or book chapter	40
		Conference paper	19
		Thesis	4
		Preprint	6
		Not a review	53
		Irrelevant topic or focus	4
		Not accessible	1
		Non-English	1
	Title/abstract screening	**Irrelevant topic or focus/no access**	**38**
		Irrelevant topic or focus	36
		Not accessible	2
	Full-text screening	**Irrelevant topic or focus**	**25**
		Insufficient depth and scope	2
		Irrelevant topic or focus	13
		Not a review	10

### Data collection process and data items

The dataset consisted of review articles identified through the search strategy. A total of 49 full-text review articles were included in the mapping review: 19 from Phase 1 and 30 from Phase 2. All of the included articles were converted from PDF to plain text format by removing images, tables, and other non-textual elements. PR performed this conversion manually to ensure accuracy. The plain text files were saved in a computer folder and imported into ATLAS.ti for qualitative coding and thematic grouping, as well as for computational analyses, such as topic modeling and clustering, using the Machine Learning for Language Toolkit (MALLET), Statistical Package for the Social Sciences (SPSS), and Python scripts.

The data items were subsequently extracted into a structured Excel spreadsheet (see Appendix 3). The following information was recorded by PR for each included review: abstract, bibliographic details (author(s), year, journal, and DOI), review type (meta-analysis or systematic, scoping, narrative, integrative, or umbrella review), thematic cluster and dominant topic (derived from latent Dirichlet allocation [LDA] and hierarchical cluster analysis [HCA]), technologies discussed, focus of the review, and main findings. These data items are summarized in Appendix 3. Qualitative interpretations were based on the emphasis placed on dominant topics within each cluster (see Figure 5, presented later in the Hierarchical clustering analysis section). Topics with the highest mean emphasis values were considered dominant, and excerpts were selected based on their alignment with the cluster's dominant topics. Examples of excerpts from Cluster 2 (immersive surgical training and healthcare simulations) include Topics 1 and 3: “Provides training in orthopedic surgical skills by recognizing anatomical structures and performing fluoroscopic techniques and surgical sutures, as well as access to various surgical environments via a 360° camera” and “Through interactive 3D environments, patients can engage in real-time discussions with healthcare providers, facilitating a more personalized and engaging experience than traditional telemedicine platforms.” Appendix 3 does not include the full set of qualitative excerpts due to their extensive volume (49 summaries × multiple supporting quotes). Instead, representative summaries are provided in the main text.

### Risk of bias assessment and effect measures

Currently, there is an absence of a tool that can be utilized to evaluate the risk of bias in mapping reviews.^63^ Consequently, a risk-of-bias assessment was not conducted, as the objective of mapping reviews is to provide a descriptive and thematic overview of the evidence landscape rather than to evaluate the effectiveness of interventions or the methodological rigor of individual studies. Consequently, the findings should be interpreted as thematic trends rather than as indicators of intervention reliability or effectiveness. Moreover, this mapping review did not encompass a meta-analysis or quantitative synthesis. Consequently, no effect measures were computed. This approach is consistent with published recommendations for mapping reviews.^52,56^ However, to improve interpretability, we conducted a descriptive assessment of certain quality-related aspects. Although several reviews—particularly narrative and nonsystematic reviews—lacked transparent reporting of article selection procedures and methodological limitations, many demonstrated strong thematic relevance, clearly defined aims, and coherent synthesis of metaverse-related content. To contextualize publication venues, we assessed journal quality (see Appendix 3) using Scimago Journal Rank (Q1–Q4). These observations were treated as findings rather than as inclusion criteria and were used to support interpretive clarity.

### Synthesis methods

**Figure 3.**
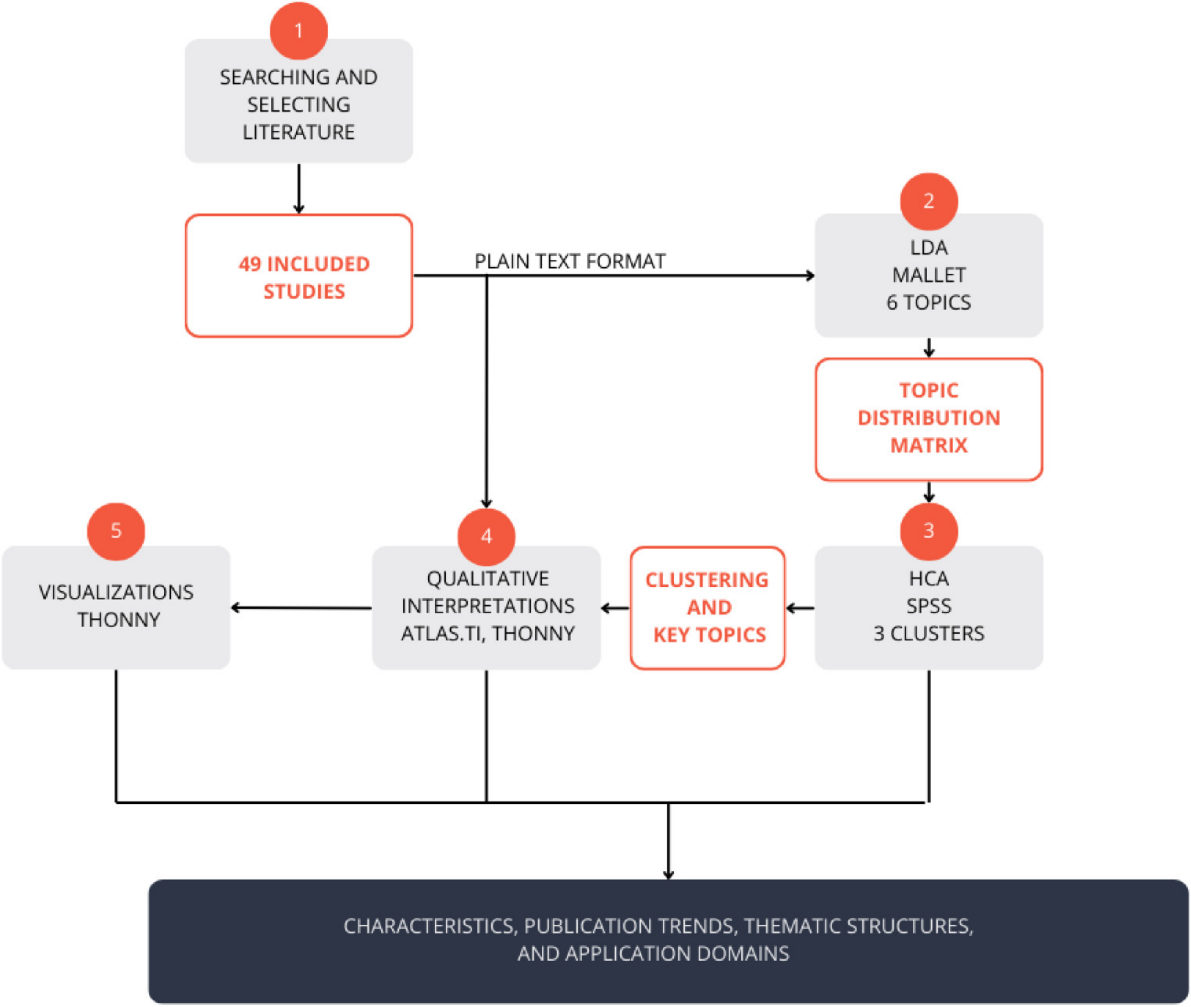
The mapping review process.

We mapped the thematic structure of the research using computational analysis and qualitative interpretation. Using LDA,^64,65^ HCA,^66^ and Python scripts (executed via THONNY https://thonny.org/ environment), we analyzed the textual content and bibliographic metadata to create visualizations and derive analytical insights. The outputs of these analyses formed the basis for qualitative interpretation.^67^ Visualizations included principal component analysis (PCA)-based scatter plots, heatmaps, top-N charts, and multipanel comparisons. This approach was useful for identifying the contours of existing knowledge and the areas in which research was concentrated. Figure 3 provides a visual representation of the synthesis process. The subsequent sections offer a more comprehensive explanation of the methodologies.

#### Topic modeling using LDA

Topic modeling was conducted using MALLET, which provides an efficient Gibbs sampling–based implementation of LDA for uncovering latent thematic structures in large text corpora.^64^ The analysis was based on machine-readable plain text versions of the articles. We used the *import-dir* command with the *keep-sequence* option to maintain word order. We also used the *remove-stopwords* option and relied on MALLET's built-in list of common English stopwords. In addition, we extended the stopword list with typical terms found in scientific and review articles (see Appendix 4, Table 1), created by analyzing word frequency distributions in Atlas.ti to identify highly frequent but noninformative terms. Domain-specific scientific terminology was retained to preserve semantic integrity. No further preprocessing steps, such as stemming or lemmatization, were applied to MALLET input (see Appendix 4, Table 2).

Multiple topic models were tested, including configurations with 1000 iterations and regular hyperparameter optimization. The full LDA parameters and preprocessing details are shown in Appendix 4. Models with six and eight topics were trained for 1000 iterations with automatic hyperparameter optimization (α optimized every 10th iteration, β set to MALLET's default of 0.01). A fixed random seed was not set in MALLET. Therefore, the results reflect MALLET's internal randomization and may show minor variation across runs. Coherence metrics were not computed for model selection, since MALLET does not provide coherence scores by default. The number of topics was determined based on topic clarity, consistency across model runs, and the balance of topic weights. Models with highly skewed topic distributions were considered less representative. The final number of topics (*n* = 6) was selected based on interpretability and balanced topic weights. The six-topic configuration offered the most balanced distribution of topic weights while preserving semantic clarity and comprehensive coverage of key themes, making it the optimal choice based on interpretability, relevance, and clear topic separation. Topic weights (in parentheses) indicate the relative prominence of each topic in the corpus.

**Topic 1** *Immersive surgical training* (0.62944) education medical learning training metaverse surgical clinical simulation skills educational nursing practice care learners medicine teaching patient web surgery traditional

**Topic 2** *Social and experiential dimensions of the metaverse* (0.87352): metaverse applications learning experiences blockchain immersive services avatars internet human computing physical software security worlds system intelligence augmented interaction iot

**Topic 3** *Personalized healthcare in the metaverse* (1.07146): metaverse immersive environments healthcare experiences integration training mental rehabilitation physical applications ethical personalized cognitive enhancing engagement privacy patient tracking interactive

**Topic 4** *Metaverse technology integration into treatment and care* (0.65986): healthcare medical patients patient devices wearable immersive care blockchain applications treatment disease surgery doctors’ system remote monitoring diagnosis smart pandemic

**Topic 5** *Metaverse in therapy and rehabilitation* (0.28813): mental metaverse anxiety treatment patients body disorders therapy augmented care physical HoloLens brain life system training symptoms show disorder depression

**Topic 6** *Metaverse in healthcare education* (0.31703): metaverse healthcare education network educational applications learning humanization collaboration growth metaverse-related

#### Hierarchical clustering analysis

The topic distribution matrix produced by MALLET was used as input for cluster analysis in SPSS. Each document was represented as a vector of topic probabilities, and these vectors were clustered via hierarchical cluster analysis using Ward's method with squared Euclidean distance, which minimizes the total within-cluster variance at each step of the agglomeration process. Since LDA does not provide a hierarchical structure, and Ward's method is not specifically designed for thematic text modeling,^68^ these methods were used in a complementary manner to enhance the overall analysis. LDA provided a probabilistic representation of thematic content, while Ward's method enabled the identification of interpretable hierarchical groupings based on topic distributions. Despite its age, Ward's method performs surprisingly well compared to many more recent approaches,^68^ making it a suitable choice when interpretable and hierarchical structures are desired.

**Figure 4.**
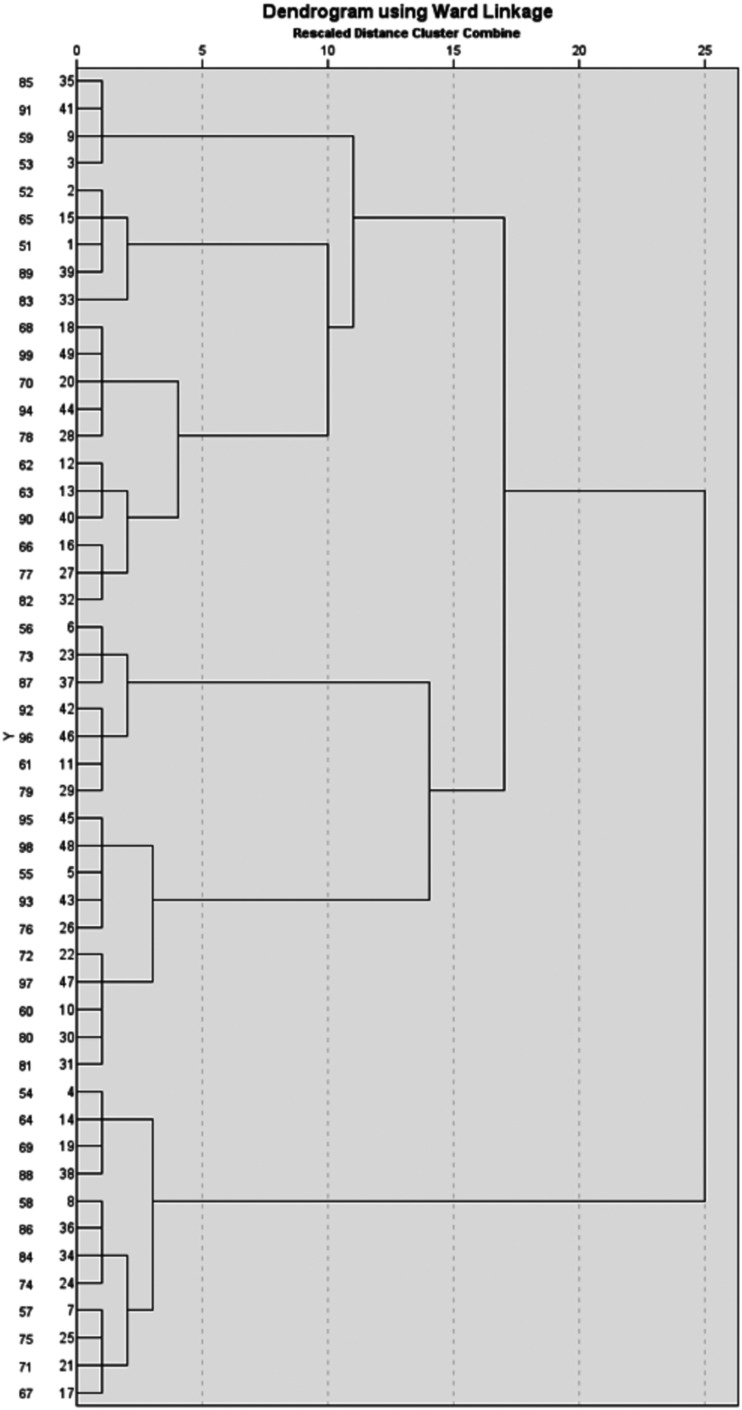
The dendrogram used to determine the optimal number of clusters.

Ward's method was selected not only for its strong empirical performance in text clustering tasks^68^ but also because it produces a dendrogram that supports the flexible and interpretable exploration of cluster structures, especially in cases where the optimal number of clusters is not known in advance. The agglomeration schedule and dendrogram were analyzed to identify the optimal number of clusters (Figure 4). The structure of the dendrogram was examined to identify substantial jumps in linkage distance, which suggest meaningful separations between clusters. Based on visual inspection, three-cluster and two-cluster solutions were considered. Both solutions were explored using hierarchical clustering and evaluated through an agglomeration schedule and a dendrogram. The agglomeration schedule showed large jumps at stages 43–48, indicating mergers of heterogeneous clusters. The largest jumps (stage 46) were retained (*k* = 3). See Appendix 5 and Tables 1 and 2 for coefficient jumps. The three-cluster solution showed a large difference (see Appendix 5, Table 3) for Topic 1 (η^2^ = .85), moderate differences for Topics 2–5 (≈.24 – .40), and a small-to-moderate effect (η^2^ = .16) for Topic 6. Levene's tests indicated heterogeneity (Appendix 5, Table 3); therefore, Welch's ANOVA was conducted alongside one-way ANOVA to confirm significant differences across clusters. Post hoc Tukey's honest significant difference (HSD) comparisons (see Appendix 5, Table 3) were consistent with the thematic interpretation. Cluster 2 placed significant emphasis on Topic 1, while Cluster 1 placed significant emphasis on Topics 2 and 5. Finally, Cluster 3 demonstrated a focus on Topics 3 and 4.

A Pearson's chi-square test of independence (see Appendix 5, Table 4) was employed to examine the association between cluster membership and dominant topics from topic modeling. A Monte Carlo exact significance (10,000 samples) was employed to enhance the reliability of the test, given that numerous anticipated cell counts were less than five. Furthermore, Likelihood-ratio chi-square test, and Fisher—Freeman—Halton exact test was computed. None of the global association tests indicated significant relationships (Appendix 5, Table 4). Although the global association tests indicated no overall relationship between topics and clusters, the linear-by-linear association χ^2^ test revealed clear monotonic patterns for several topics. According to the trend statistics (Appendix 5, Table 4), significant linear-by-linear associations were identified for Topics 2, 3, 4, 5, and 6, indicating systematic directional variation across clusters. In conjunction with the dendrogram structure (see Figure 4) and significant linear-by-linear trends, this suggests that the three-cluster solution is statistically supported and thematically interpretable.

**Table 4.** Included articles and clusters.

Cluster	Included articles
**Cluster 1:** *Immersive therapeutic and educational applications with intelligent integration*	Aboul-Yazeed et al. (2025),^69^ Agac et al. (2025),^70^ Bansal et al. (2022),^71^ Cerasa et al. (2022),^72^ Enamorado-Díaz et al. (2025),^73^ Fadhel et al. (2024),^74^ Gonzalez-Moreno (2023),^75^ Gruson et al. (2023),^76^ Jain et al. (2024),^77^ Kataria et al. (2023),^78^ Li et al. (2024),^79^ Naqishbandi et al. (2023),^80^ Musamih et al. (2022),^81^ Nguyen & Voznak (2024),^82^ Pillay et al. (2024),^83^ Rejeb et al. (2023),^84^ Sharma et al. (2024),^85^ Sönmez & Hocaoğlu (2024),^86^ Ullah et al. (2023),^87^ Bhugaonkar et al. (2022)^88^
**Cluster 2:** *Immersive technologies for surgical training and clinical simulation*	Bernardes et al. (2024),^89^ Burlacu et al. (2025),^90^ Capasso et al. (2025),^91^ Ferorelli et al. (2025),^92^ Ibrahim et al. (2025),^93^ Jauniaux et al. (2025),^94^ Kayaalp et al. (2025),^95^ Lau et al. (2025),^96^ Lewis et al. (2024),^97^ Ogundiya et al. (2024),^98^ Popov et al. (2024),^99^ Randazzo et al. (2023)^100^
**Cluster 3:** *Integrated immersive and intelligent technologies for personalized and networked healthcare*.	bin Zainuddin et al. (2024),^101^ Buragohain et al. (2025),^102^ Chengoden et al. (2023),^103^ Daneshfard et al. (2025),^104^ Khan et al. (2024),^105^ Kourtesis (2024),^106^ Li et al. (2025),^107^ Morone et al. (2025),^108^ Mozumder et al. (2023),^109^ Murala et al. (2023),^110^ Raman et al. (2024),^111^ Tang et al. (2024),^112^ Turab & Jamil (2023),^113^ Vahdati et al. (2025),^114^ Vecchio et al. (2025),^115^ Zhang et al. (2024),^116^ Żydowicz et al. (2024)^117^

**Figure 5.**
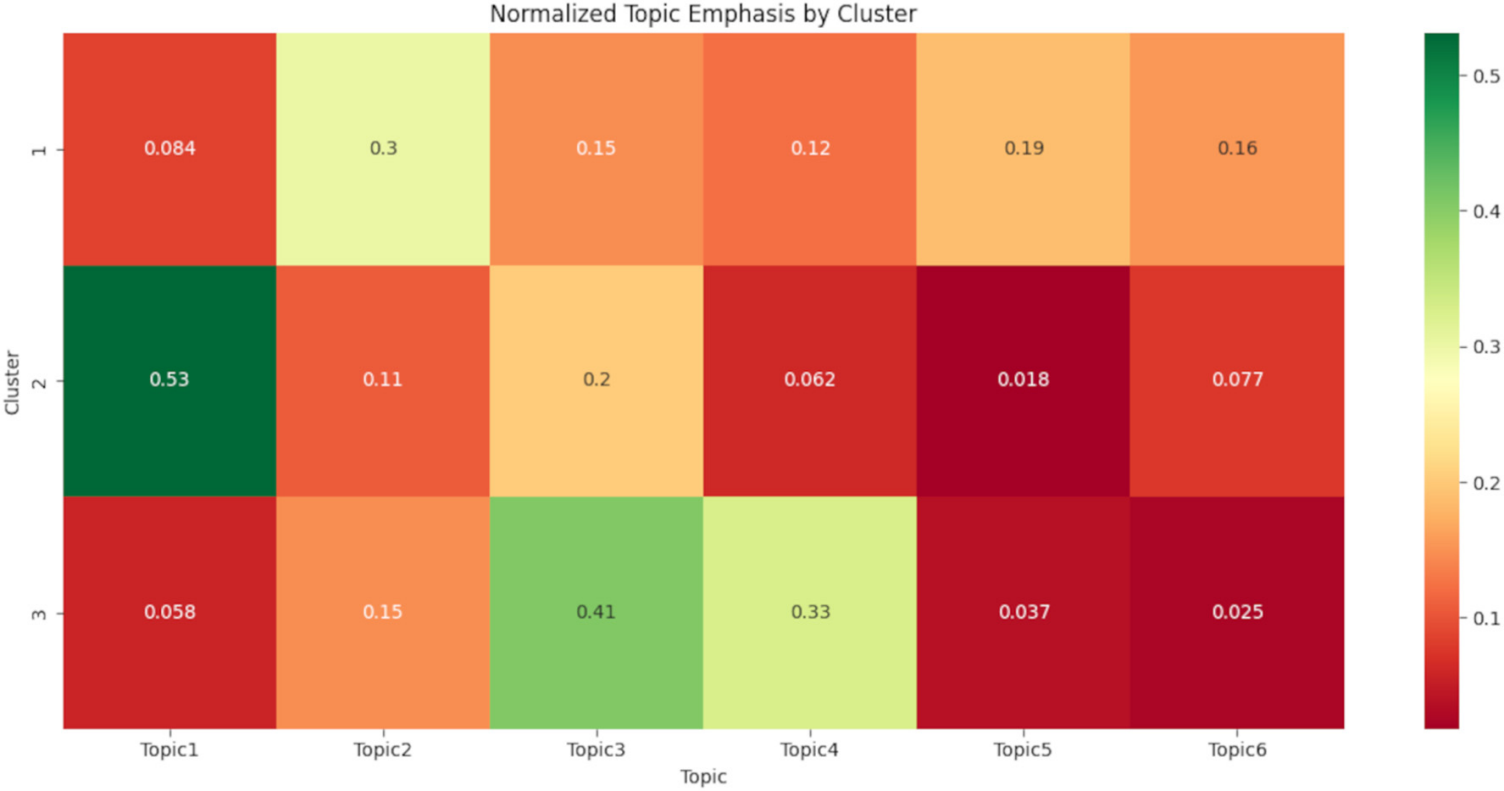
Heatmap of topic emphasis by cluster.

Figure 5 illustrates the relative emphasis of six topics across the three thematic clusters identified through hierarchical clustering. In Figure 5, each row represents a cluster, and each column corresponds to one of the six topics. The values in the cells indicate normalized weights, where each row sums to 1, showing the proportion of each topic within a cluster. Darker green shades indicate higher emphasis, while red shades indicate lower emphasis. Cluster 2 strongly emphasizes Topic 1 (*immersive surgical training*; 0.53). Cluster 3 emphasizes Topic 3 (*personalized healthcare in the metaverse*; 0.41) and Topic 4 (*metaverse technology integration into treatment and care*; 0.33) the most. Cluster 1 is more balanced, but Topic 2 (*social and experiential dimensions of the metaverse*) and Topic 5 (*metaverse in therapy and rehabilitation*) are slightly stronger.

**Figure 6.**
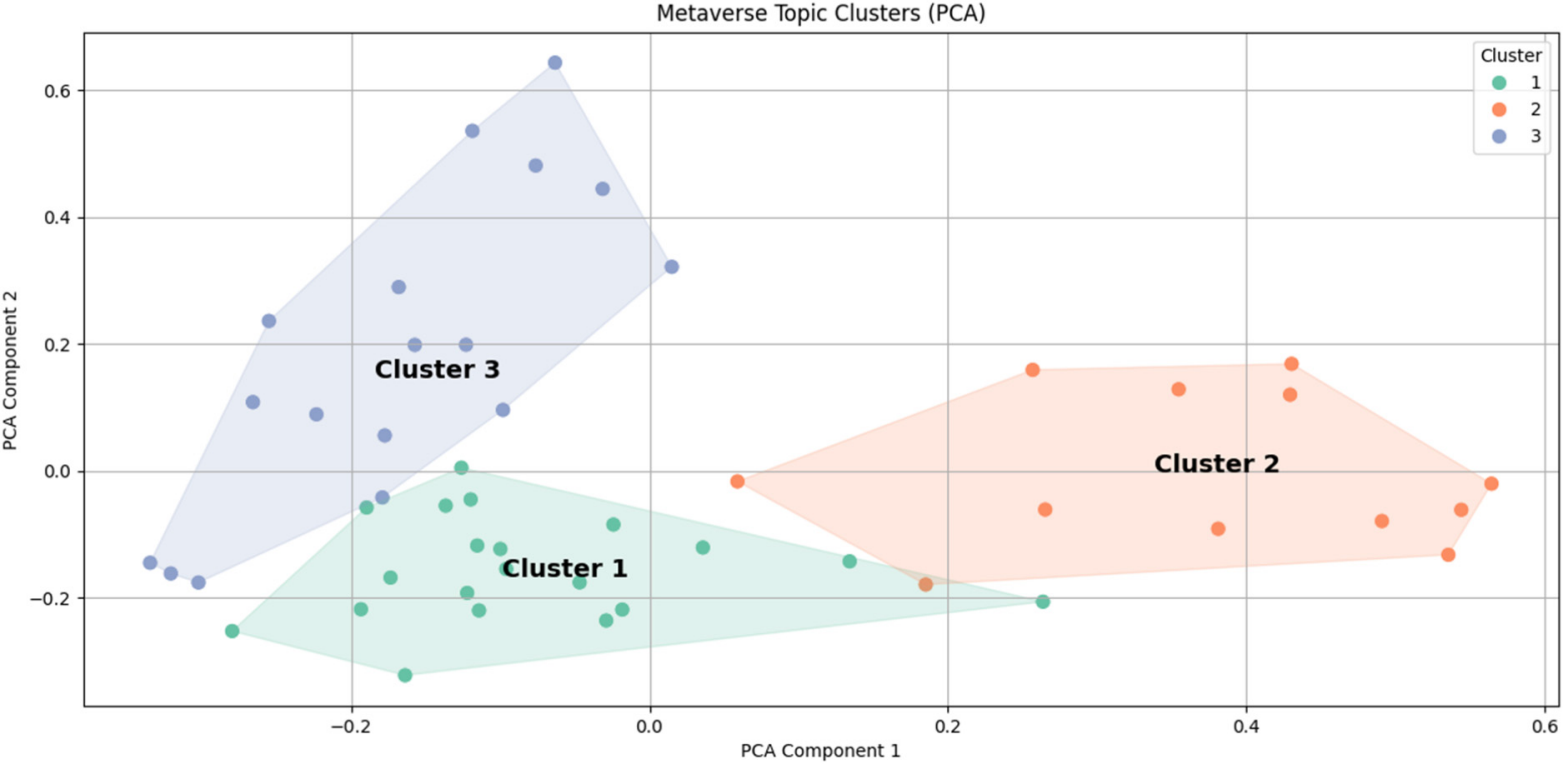
PCA-based visualization of the topic distribution matrix.

To complement the heatmap and dendrogram, we generated a PCA-based visualization of the topic distribution matrix (see Figure 6). PCA was applied after hierarchical clustering to provide a two-dimensional representation of the thematic relationships. In Figure 6, each point represents a review article, positioned according to its two principal components derived from the six MALLET topics. Colors indicate cluster membership (Clusters 1–3), and convex hulls outline cluster boundaries. PCA was performed in Python (Thonny IDE) using *scikit-learn* to visualize topic distributions after hierarchical clustering. The first two components explained 57.6% of the variance (PC1 = 32.6%, PC2 = 25.1%). The first principal component (PC1) was primarily influenced by *immersive surgical training in the metaverse* (loading = 0.8191) and negatively by *metaverse technology integration into treatment and care* (loading = −0.4433), indicating a contrast between training-focused and technology integration-focused articles. The second component (PC2) was most strongly associated with *personalized healthcare in the metaverse* (loading = 0.8699) and negatively associated with *social and experiential dimensions of the metaverse* (loading = −0.3972), suggesting a thematic axis between personalized healthcare and social/experiential aspects.

Overall, the PCA plot provides a spatial overview of the thematic relationships among the clusters. Cluster 2 is positioned toward training and simulation themes, Cluster 3 aligns with personalized healthcare and integration, and Cluster 1 leans toward social and therapeutic dimensions. The cluster names were derived from a qualitative interpretation of the dominant LDA topics and supported by PCA loadings: *immersive therapeutic and educational applications with intelligent integration* (Cluster 1), *immersive technologies for surgical training and clinical simulation* (Cluster 2), and *integrated immersive and intelligent technologies for personalized and networked healthcare* (Cluster 3). Table 4 summarizes the included studies and assigned clusters.

#### Qualitative interpretation

After topic modeling and hierarchical clustering, all included reviews were grouped by cluster, using ATLAS.ti, and read in full. We adopted a topic-guided qualitative interpretation strategy inspired by Romero et al.,^67^ who demonstrated the value of human validation in topic modeling to support qualitative analyses. One author (PR) prepared thematic summaries for each cluster based on their alignment with dominant topics within each cluster; these dominant topics were determined using normalized topic emphasis scores (see Figure 5). Topics with the highest mean emphasis values were considered dominant.

To facilitate consistency checking during the qualitative interpretation stage, an AI-assisted agreement check (Microsoft Copilot) was employed. This approach is consistent with evolving qualitative research methodologies and is promising.^118^ Copilot was provided exclusively with text excerpts and topic labels specific to the cluster, without the accompanying bibliographic metadata. The tool generated a summary based on this limited input, and this was compared with a human-produced summary to assess agreement in topic coverage, thematic alignment, terminology, and emphasis. The AI suggestions functioned exclusively as prompts for human re-checking, and no AI-generated text, interpretations, or syntheses were incorporated into the analysis or the manuscript. All qualitative interpretations, coding decisions, and thematic synthesis were conducted by human researchers. Confidentiality was ensured by utilizing the university's enterprise Copilot environment.

All clusters had good agreement (>80%). The comparison process was repeated multiple times, and human interpretation remained primary. To ensure independence between iterations, Copilot's chat memory was cleared before each validation. This prevented previous outputs from influencing subsequent comparisons. We noted minor differences in narrative style and level of detail, with the human summaries offering richer contextualization. In some cases, Copilot's suggestions prompted us to revisit the source material for clarification. Final interpretations and all thematic decisions remained human-driven. Copilot enabled a structured, impartial, and efficient agreement-checking workflow. This step ensured semantic alignment and thematic coherence before any complementary computational text analysis was conducted.

Subsequently, three complementary analyses—N-gram analysis, promise vs. evidence–signal analysis, and risk category analysis—were performed on machine-readable plain-text versions of the reviews. These complementary analyses yielded signals providing a quantitative basis for triangulating the qualitative interpretations. The analyses were implemented in Python (Thonny IDE). All textual data underwent a unified preprocessing pipeline to ensure comparability across clusters and analyses. Initially, the raw text files underwent a series of transformations to ensure data integrity and consistency. Specifically, they were first converted to lowercase, and nonalphanumeric characters were removed, with hyphens preserved to maintain contextual integrity. In addition, whitespace was collapsed, and numeric tokens were filtered out where appropriate, ensuring the accuracy and relevance of the data. To reduce noise and improve semantic clarity, an extended stopword list was applied in addition to standard English function words. The list encompassed frequent yet noninformative academic terms, such as “analysis,” “method,” “study,” “review,” “paper,” “research,” and “results.” The vocabulary employed encompassed structural words—such as “section,” “title,” and “topics”—and generic verbs—including “allow,” “enable,” “provide,” and “include.” The tokenization process was executed through the utilization of whitespace segmentation.

For N-gram analysis, bigrams were generated from a set of tokens that had been filtered to remove stop words. This was followed by a series of steps involving regex-based phrase normalization (e.g., plural → singular) and abbreviation unification for XR-, AI-, and DT-related terms. The order variants were consolidated. The rankings were derived from two distinct metrics: normalized frequency (per 1000 words) and pointwise mutual information. In the context of promise versus evidence scoring, a less stringent normalization procedure was implemented with the objective of preserving statistical markers (e.g., *P* ≤ .05, 95% CI) and numeric values deemed essential for evidence detection. Lexicon-based weighting was employed to categorize documents as promise-leaning, balanced, or evidence-leaning. Risk-related themes were initially identified through a qualitative analysis and word frequency distributions in ATLAS.ti, whereby the most prevalent risk categories cited in the reviews were systematically coded. These categories—privacy and security, ethics and governance, accessibility and equity, technical and performance—formed the basis of a predefined taxonomy. Subsequently, quantitative frequency analysis was performed in Python using precomputed tables normalized per 1000 words. The visualizations encompassed cluster-level bar charts.

## Results

We mapped 49 review articles to identify the metaverse's thematic clusters and application domains in healthcare (see Appendix 3). Most of the reviews were published recently, with the majority appearing in 2024 (*n* = 18) and 2025 (*n* = 16), which reflects the growing academic interest in this topic. General reviews dominated the dataset (*n* = 25), while systematic reviews (*n* = 9) were less frequent but generally more rigorous and were published in higher-ranked journals. The reviews spanned a diverse range of journals, from *IEEE Access* and *Cureus* to *Heliyon* and *Frontiers in Medicine*, highlighting the broad relevance of metaverse technologies across disciplines. The following section explores the thematic clusters and application domains identified through topic modeling and hierarchical clustering.

### Cluster-level synthesis

**Figure 7.**
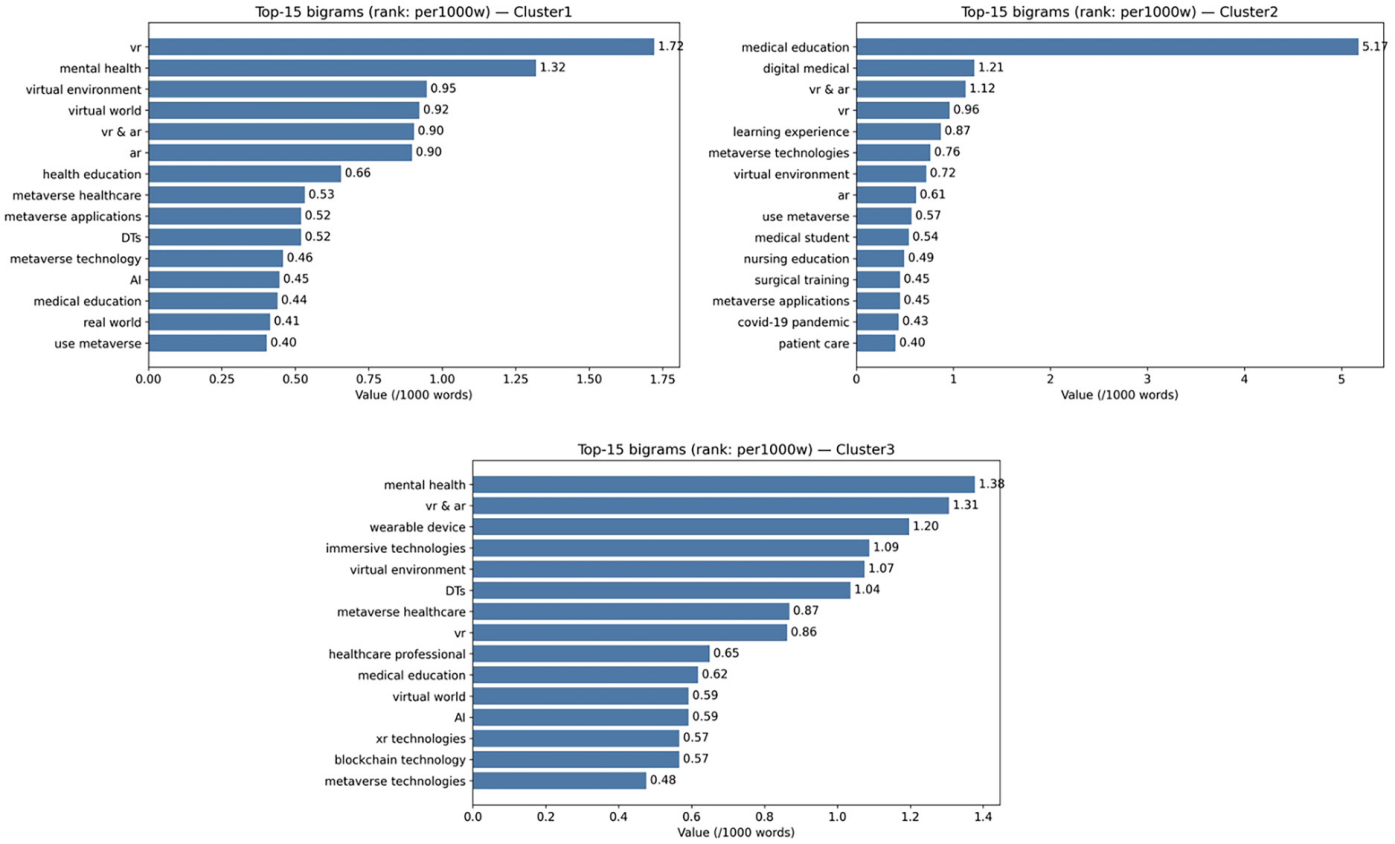
Top bigrams by normalized frequency across the three clusters.

**Figure 8.**
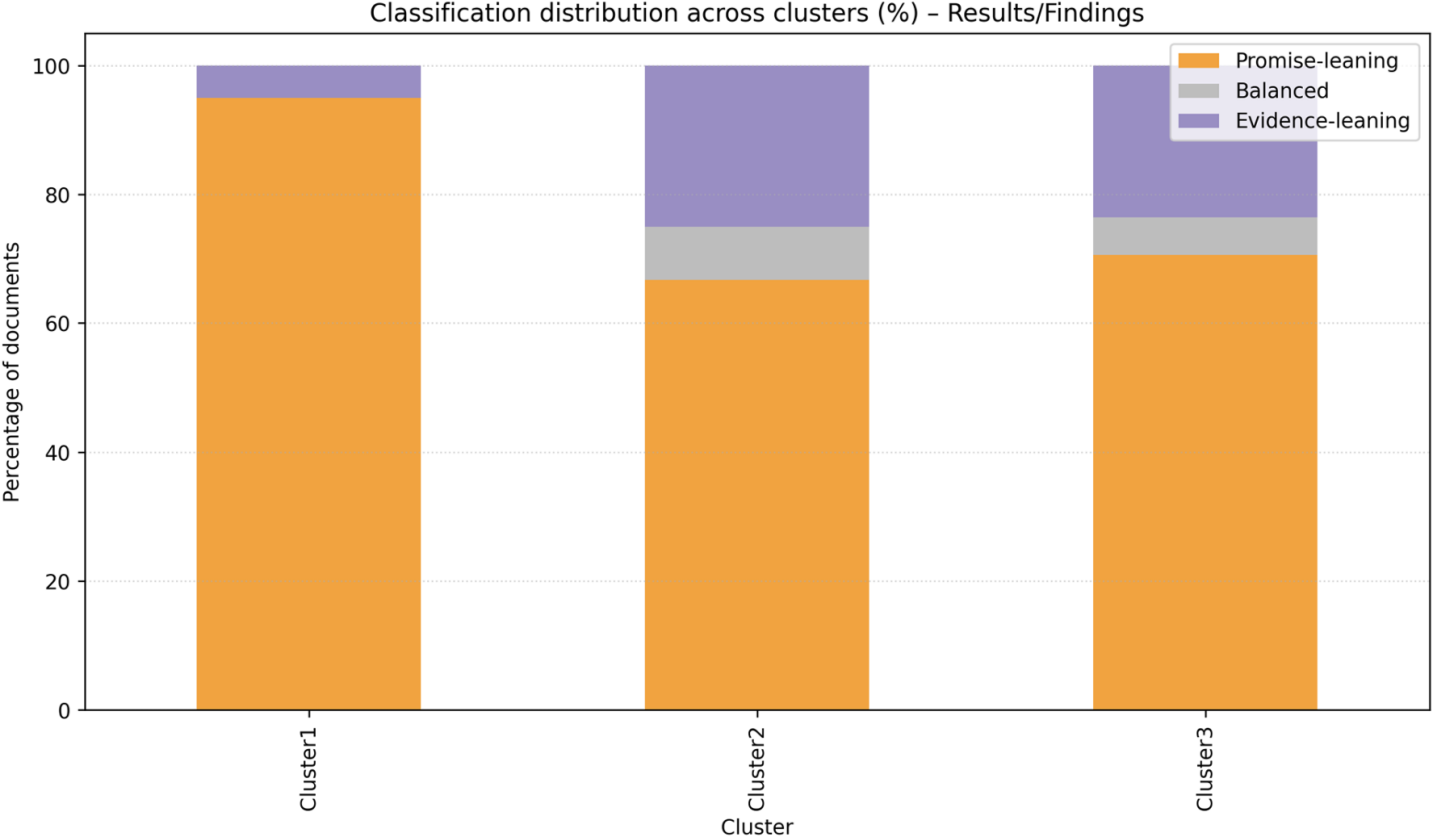
Distribution of promise vs. evidence signals across clusters (%).

**Figure 9.**
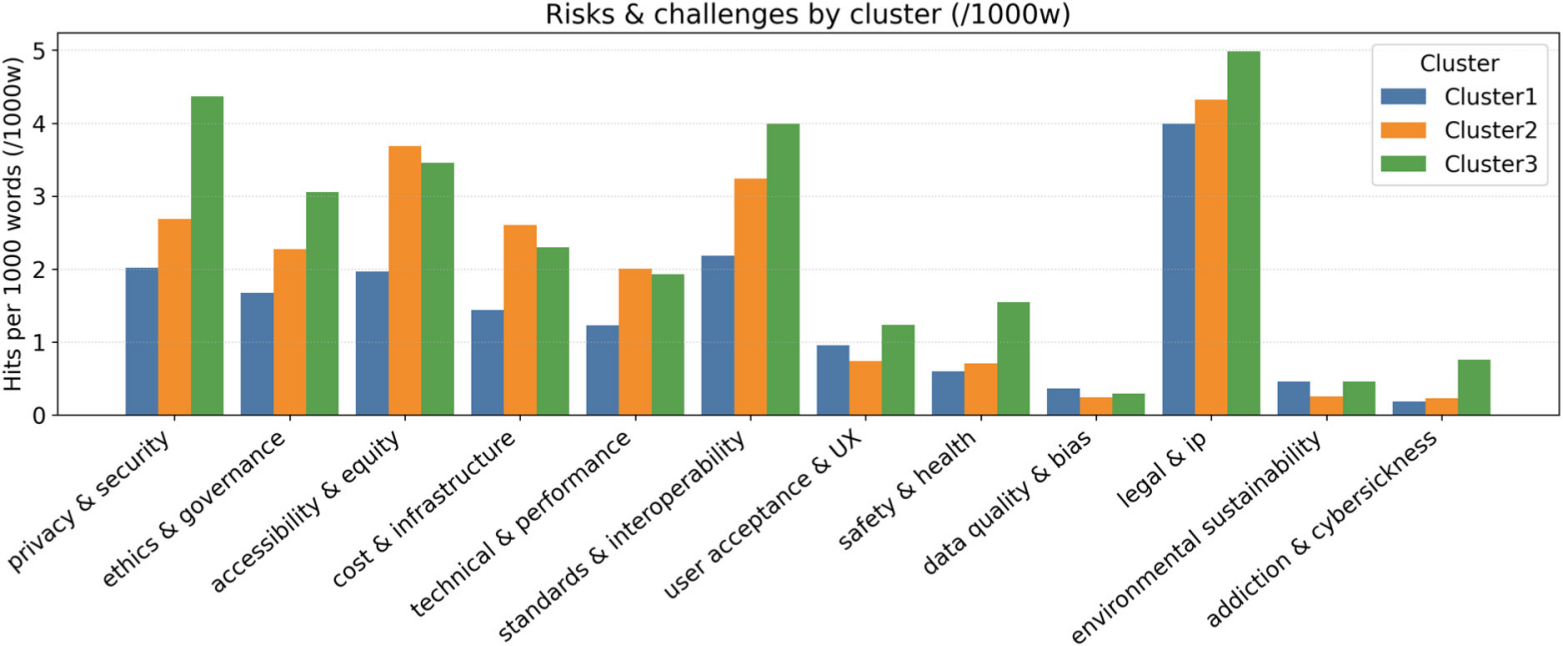
Risk and challenge frequencies across clusters.

The predominant focus of Cluster 1 was general healthcare, with subthemes pertaining to mental health and healthcare education, as illustrated in Table 5. A closer examination of bigram profiles indicated a pronounced emphasis on mental health, virtual environments, and health education (see Figure 7). Most of the reviewed articles demonstrated a focus on promise-leaning outcomes, suggesting an emphasis on preliminary conceptualization rather than validated results (Figure 8). The risks emphasized included privacy, ethics, and governance, alongside interoperability challenges (Figure 9). Cluster 2 centered on healthcare education and surgical specialties, as illustrated in Table 5. As shown in Figure 7, the bigram signals encompassed medical education, surgical training, and learning experiences. Evidence indicated that posture was more balanced than in Cluster 1, as demonstrated by practical deployments in simulation (see Figure 8). The identified barriers encompassed financial constraints, infrastructural limitations, and legal and intellectual property considerations (see Figure 9). Cluster 3, in turn, focused on specialized domains, such as antiaging and nuclear medicine, and the combination of XR, AI, and DTs to enable personalized remote care models and chronic care (see Table 5). Bigram patterns were exhibited in wearable devices, DTs, and immersive technologies (Figure 7). Notwithstanding the integrative scope, the phenomenon was presented more in terms of its promise than current evidence supports (see Figure 8). The risks under discussion were extensive, encompassing privacy, standards, data quality, and sustainability (see Figure 9).

**Table 5.** Cluster-level summary.

Cluster	Typical focus	Key technologies	Main finding	Representative studies
**1. Immersive therapeutic and educational applications with intelligent integration (*n*** **=** **20)**	HC (*n* = 8), MA (*n* = 4), DS (*n* = 3; e.g., laboratory medicine, longevity), MH (*n* = 3)	XR (*n* = 19), AI (*n* = 16), DTs (*n* = 14), IoT (*n* = 11), cloud (*n* = 10)	The metaverse provides controlled, personalized therapeutic and rehabilitative environments that improve psychological and physical outcomes, empower patients through active engagement and personalized care, and enhance education with immersive, hands-on experiences. However, widespread adoption is hindered by privacy, safety, and integration challenges.	e.g., Aboul-Yazeed et al. (2025; mental-health therapy),^69^ Bansal et al. (2022; adherence and engagement),^71^ Pillay et al. (2024; physical rehabilitation)^83^
**2. Immersive technologies for surgical training and clinical simulation (*n*** **=** **12)**	HE (*n* = 8), DS (*n* = 4; e.g., surgery, orthopedics, urology, forensic medicine)	XR (*n* = 12), AI (*n* = 9), haptics (*n* = 3), wearables (*n* = 3), DTs/robotics (*n* = 2)	The metaverse enables realistic, risk-free surgical training and clinical simulations, supporting skills development, collaborative learning, and real-time feedback, while improving technical proficiency, decision-making, and teamwork beyond traditional methods. However, evidence in acute care is limited, and adoption faces cost, privacy, and ethical barriers.	e.g., Ibrahim et al. (2025; error reduction),^93^ Jauniaux et al. (2025; skills transfer),^94^ Kayaalp et al. (2025; skills transfer),^95^ Popov et al. (2024; retention)^99^
**3. Integrated immersive and intelligent technologies for personalized and networked healthcare (*n*** **=** **17)**	DS (*n* = 13; e.g., antiaging, chronic conditions, nuclear medicine), HC (*n* = 3)	XR (*n* = 16), AI (*n* = 14), DTs (*n* = 12), wearables (*n* = 9), IoT (*n* = 9)	The metaverse integrates digital twins, wearables, and immersive environments to deliver personalized care, remote monitoring, and patient engagement, enhancing clinical decision-making, treatment planning, education, and overall healthcare accessibility. However, validation, privacy, cost, and regulatory gaps remain major obstacles.	e.g., Chengoden et al. (2023; remote monitoring platforms),^103^ Raman et al. (2024; care pathway integration),^111^ Vahdati et al. (2025; hospital-level digital twin)^114^

Focus codes: MH = mental health; HE = healthcare education; HC = healthcare (general); DS = domain-specific; MA = metaverse applications.

### Detailed thematic descriptions and evidence examples

Building on the cluster-level synthesis, this section provides detailed thematic descriptions and representative evidence from the included reviews.

#### Cluster 1: Immersive therapeutic and educational applications (*n* = 20)

Cluster 1 encompassed diverse topics yet consistently centered on the therapeutic and rehabilitative uses of the metaverse in XR interventions. These kinds of interventions provide controlled, individualized environments under therapist supervision.^72^ Therapeutic interventions addressed exposure therapy for anxiety, phobias, posttraumatic stress disorder (PTSD), depression, and pain management^71,72,86^ as well as behavioral therapies targeting motivation, attention, and social skills.^72,77,86^ Rehabilitation interventions, in turn, addressed poststroke motor recovery, support for neurodegenerative disorders, Parkinson's gait training, and cognitive functions in dementia.^76,78,86,99^ The emerging approaches discussed included brain–computer interfaces for paralysis^79^ and multisensory regenerative virtual therapy.^72^ Aggregated evidence from multiple reviews indicated that metaverse-based interventions, at their best, can improve both psychological and physical outcomes. For example, physical activity increased and depression and anxiety decreased in metaverse groups compared to baseline.^69^ VR exposure therapy substantially reduced PTSD symptoms (90%) and depression (83%), while acceptance-based virtual exposure lowered social anxiety and avoidance.^86^ VR therapy demonstrated efficacy in addressing acrophobia and social anxiety disorder, while AR interventions showed promise in enhancing fundamental skills and motivation in individuals with autism.^86^ Nature-based VR experiences reduced stress and anxiety,^83^ and virtual worlds such as Second Life alleviated depression and social isolation, improving quality of life.^86^ Rehabilitation evidence included VR improving balance, gait, and motor function in Parkinson's disease (12 RCTs, *n* = 360).^78^ Although these findings are promising, effectiveness remains insufficiently defined, and cost–benefit analyses are lacking.^72^

Beyond therapeutic and rehabilitative uses, the metaverse supports patient empowerment through personalized care pathways, DTs, and immersive environments that promote active engagement and self-management.^71,77,79,81^ Patients can access individualized health insights and connect with peers or providers in virtual spaces.^77,79,81^ The metaverse allows patients and clinicians to meet in the same virtual or physical reality, facilitating better tracking of compliance and treatment options for specific patients.^75^

Educational uses include interactive anatomy and simulated clinical procedures.^70,71^ The goal is to create virtual spaces in which social interaction and collaboration are more immersive and overcome traditional communication limits.^73^ The metaverse has the potential to enhance students’ engagement and learning outcomes by providing authentic, hands-on experiences unavailable in traditional classrooms.^84^ Educational metaverse environments were shown to increase students’ motivation and technology acceptance.^70^

Despite these benefits, major challenges persist. These include privacy and data security,^69,74,76,82,87^ ethical concerns,^78,81^ high costs,^69,81–83^ accessibility barriers,^73,78,79,85^ regulatory gaps,^78,82,84,87^ interoperability issues, and lack of standardization.^73,76,81,87^ Addressing these issues requires robust governance, ethical frameworks, and interdisciplinary collaboration.^73,84^

#### Cluster 2: Immersive technologies for surgical training and clinical simulation (*n* = 12)

Cluster 2 positioned the metaverse as a transformative tool for medical education and professional training. Immersive technologies support realistic surgical simulations, distributed collaborative learning, and immediate performance feedback.^90,92,94,96,97^ For instance, AR simulations are being used in orthopedic trauma surgery training to offer versatile, patient-specific scenarios.^90^ AR was utilized to project virtual images onto physical models and cadavers to facilitate the acquisition of supplementary insights and annotations during the course of dissections.^97^ Immersive technologies in the metaverse allow virtual immersion into the human body, providing students with a realistic view of anatomy and surgical approaches from remote settings.^94^ For example, a medical learner might use VR to explore the human body and then switch to AR to practice surgery on a physical model.^97^ Dangerous or high-risk activities, such as difficult surgeries, can be reproduced through these virtual simulation environments.^100^ For instance, virtual surgery simulation with haptic feedback allows learners to perform procedures in a realistic virtual setting and observe real-time results.^97^ AI could further introduce variation and simulate intraoperative complications, as in real life.^100^

Thus, the metaverse provides an engaging and immersive environment for knowledge retention and skills acquisition by simulating real-world scenarios in real time.^93^ It facilitates training by offering opportunities for the development of competencies such as the management of equipment, the execution of complex manual skills, and training on simulated patients utilizing real patient data.^89^ Additional potential uses include virtual workshops, lectures, patient communication training,^89,96,100^ and equity-enhancing solutions, such as safe practice environments for pregnant surgeons.^91^ For instance, the metaverse was employed for training purposes—specifically in the form of an online workshop that facilitated the observation of robotic liver resection procedures by surgeons from various global locations.^94^ Furthermore, immersive technologies can simulate scenarios that involve coordination among medical teams, promoting teamwork and communication skills, such as in emergencies.^97^ VR-based simulations were used in emergency medicine to improve teamwork and communication, allowing teams to practice protocols and improve response times without physical simulation centers.^90^

Evidence showed that VR-based metaverse training improves hand–eye coordination, procedural accuracy, and technical proficiency, particularly in arthroscopy and other surgical fields.^95^ In addition, studies showed that immersive learning helps people remember knowledge, technical skills, and critical thinking. For instance, VR simulator training maintained laparoscopic skills for up to 18 months, with only a small decline after six months.^99^ More research suggested that immersive environments improve skill transfer, decision-making, and confidence in the workplace.^93,94^ Evidence from two decades suggests that technology-enhanced simulation may be better at teaching clinical skills than traditional approaches.^99^ Trainees using VR often achieve higher competence and perform complex procedures more effectively than those trained using conventional methods.^95^

Despite these advantages, several challenges persist. Metaverse-based training may reduce the emphasis on core clinical competencies, such as physical examination and nonverbal communication.^90,92,100^ AI-driven simulations risk embedding biases,^92,94,100^ and many educators lack the technical expertise required for implementation.^89,90,93,97^ Evidence on long-term effectiveness and skill transferability remains limited.^93,95,99^ High costs, technical requirements, and unequal access to hardware and connectivity may exacerbate disparities.^89,90,95–98^ Sensitive patient data necessitate strong safeguards,^94,97,98,100^ and clear ethical guidelines and technical standards are needed.^92^ Addressing these barriers requires interdisciplinary collaboration among educators, technologists, clinicians, and policymakers.^93,95^

#### Cluster 3: Integrated immersive and intelligent technologies for personalized and networked healthcare (*n* = 17)

In Cluster 3, the metaverse was depicted as a multifaceted platform enabling personalized care, remote monitoring, and patient engagement through DTs, wearables, and immersive environments. These technologies have the potential to complement each other, enhancing the efficiency and productivity of various processes across different fields.^108^ Reported applications include virtual consultations,^101,104,107^ simulation-based diagnostics,^102,104,110^ immersive medical training^101,105,112 [55,72,92]^—all potentially contributing to improved clinical decision-making,^108,112^ procedural accuracy,^112,117^ and patient understanding.^103,113^

DTs support patient-specific modeling to simulate disease progression and predict treatment outcomes.^111,113,116^ When combined with AI, IoT, and wearable sensors, DTs enable continuous monitoring and proactive interventions.^111,114,116^ AI further facilitates personalized treatment planning, blockchain ensures secure data exchange,^109^ and wearables capture physiological and behavioral data for mental and physical health management.^102,106,110^ Avatars can visualize lifestyle impacts on health, enhancing patient engagement.^109^ Promising applications include virtual physiotherapy, biopsy and counseling tools, immersive rehabilitation, biofeedback systems, and 360° anatomical reconstructions for surgical planning.^102,103,108^

The healthcare sector is increasingly adopting telemedicine and remote care solutions to improve outcomes, accessibility, and provider collaboration.^101^ Some evidence indicated that digital transformation through AI, cloud computing, AR, and VR has enhanced service access and patient–clinician interactions, enabling virtual care, remote monitoring, digital diagnostics, decision support, and at-home prescription delivery.^103^ For instance, wearable technologies, including TENGs, EEG sensors, and smart textiles, enhance data accuracy and support real-time monitoring integrated with immersive environments.^110^ Metaverse technologies were also shown to support 3D patient representations for accelerated treatment planning,^103^ improve clinical trial processes, such as enrollment and monitoring, and enable secure blockchain-based storage of personal medical data for personalized care.^104^ For instance, AI-driven systems and wearables enabled continuous monitoring and personalized activity guidance for conditions such as diabetes and obesity.^110^ VR-based education was also shown to improve patient comprehension and adherence,^107^ and randomized trials reported higher satisfaction with metaverse-based home treatment than with clinical settings.^108^ A substantial body of research validated the efficacy of VR in patient empowerment, education, rehabilitation, cancer symptom management, psychiatric care, and the mitigation of treatment side effects.^117^ Immersive applications such as HoloLens-based 3D brain overlays also enhance surgical performance. For example, VR-based training improved surgical outcomes by 230% compared to conventional methods.^105^ Smart gloves improve precision in VR surgical training.^114^ Immersive simulations were proven to enhance technical and nontechnical skills,^105^ allowing for repetitive practice and thus improving muscle memory and confidence in critical skills.^106^ Therefore immersive technologies can improve patient outcomes. For instance, VR training was shown to improve hip arthroscopy performance by 83%.^105^

Despite these advances, significant challenges remain. Key concerns include data privacy and cybersecurity,^106,108,111–113^ regulatory gaps,^101,103,113^ interoperability limitations,^73,112,113^ and accessibility barriers.^101,113,115^ High costs,^102,103,105^ digital literacy gaps,^112^ and environmental sustainability issues^103,104^ further complicate adoption. Additional risks involve overreliance on virtual environments,^110,115^ user dissociation,^106^ and weakened face-to-face rapport.^103^ Therefore, responsible implementation requires multidisciplinary collaboration and user-centered design,^113^ evidence-based validation,^102,104,111,113,114,117^ and robust legal and ethical frameworks.^104,109^ When ethically integrated, metaverse technologies have the potential to support equitable, personalized, and technology-enhanced healthcare.^108,111^

## Discussion

The results of this mapping review of review articles indicate a growing interest in metaverse-related healthcare research. However, the predominance of general reviews and the limited number of systematic syntheses suggest that the field still lacks sufficient empirical depth to draw robust, evidence-based conclusions about metaverse-related outcomes in the healthcare domain. Nonetheless, the field is beginning to shift from exploratory conceptual mapping to more structured synthesis, interdisciplinary integration, and patient-centered innovation. While our focus was on articles that explicitly discussed the metaverse concept, many included reviews that tended to discuss technologies individually rather than conceptualizing the metaverse as a holistic ecosystem. Future research would benefit from clearer definitions, standardized frameworks, and more empirical studies to enable systematic synthesis.

The three thematic clusters identified in the review reflect distinct application domains but share overlapping technologies and challenges. Cluster 1 emphasizes patient-centered, immersive environments that conceptually support mental health, pain management, and rehabilitation, aligning with the WHO's strategic objective of human-centered systems (SO4).^43^ The reviews emphasized that the metaverse has the potential to offer controlled, personalized therapeutic and rehabilitative environments that improve psychological and physical outcomes, empower patients through active engagement and personalized care, and enhance medical education with immersive, hands-on learning experiences. Cluster 2 positions the metaverse as a transformative tool for medical education and professional training, aligning with the WHO's strategic objective of human-centered systems through workforce skills development, risk-free practice opportunities, and improved preparedness. The metaverse enables immersive surgical training and clinical simulation by providing realistic, risk-free environments for skills development, collaborative learning, and real-time feedback, thereby enhancing technical proficiency, decision-making, and teamwork beyond traditional methods. Cluster 3 highlights the integration of intelligent and immersive technologies into healthcare pathways, demonstrating a strong alignment with the WHO's strategic objective of strategy implementation (SO2) and human-centered systems. In this cluster, the metaverse ecosystem idea becomes more explicit; the focus shifts toward system-level integration. The metaverse has the potential to integrate personalized care, remote monitoring, and patient engagement through digital twins, wearables, and immersive environments, thereby enhancing clinical decision-making, treatment planning, and education while improving outcomes and accessibility. Across all clusters, elements of the WHO's strategic objectives of governance (SO3) and strategy implementation^43^ were evident in discussions of ethical, regulatory, and implementation challenges.

These findings are supported by earlier research demonstrating the increasing use of digital tools in diagnosis, treatment planning, and patient education.^3,16,21,23,30,31,119^ The clusters reflect a broader shift from experimental applications to scalable, system-level solutions. Prior studies have shown how immersive technologies are evolving toward more integrated and practical use cases.^6,16,18,24,29^ Overall, these trends reflect a broad technological transformation^1^ in which digital innovations are increasingly central to healthcare delivery.^2,43^

While the clusters differ in focus, they share common challenges related to the responsible integration of metaverse technologies. Ethical, regulatory, and technical concerns, such as data privacy, user safety, and equitable access, continue to surface.^37,120^ Although solutions such as blockchain and cloud-based systems offer promise,^120^ they require careful validation and may pose additional privacy threats. For instance, wearable devices may produce unreliable physiological data if external factors interfere. Robust clinical validation and inclusive design are essential to ensuring safe, effective, and trustworthy applications. This supports the notion that although the metaverse is a multifaceted ecosystem with great potential,^9,11^ continued research is necessary to identify its risks.^37^ To realize its potential, we must move beyond technology and toward interdisciplinary collaboration and user-centered design.^10^ The findings thus suggest that the metaverse holds considerable potential to steer healthcare toward more human-centered models, strengthen knowledge and skills development, and facilitate collaboration. However, the most substantial work remains in the areas of strategy implementation (SO2) and governance (SO3) to ensure that the metaverse can function as a coherent and sustainable ecosystem.

**Figure 10.**
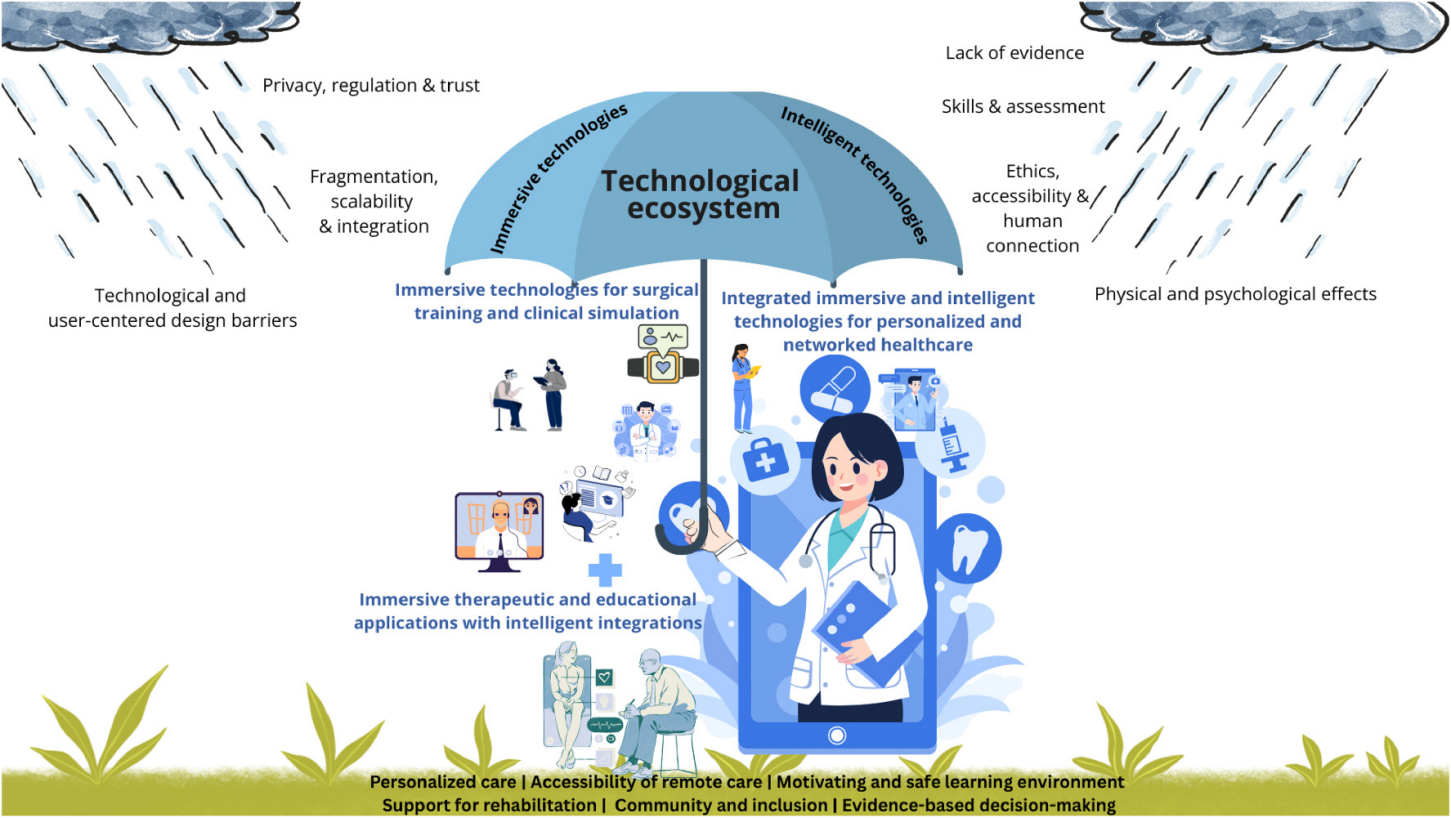
Conceptual visualization of the technological ecosystem in the healthcare metaverse, with the umbrella metaphor illustrating the interplay between technologies, users, challenges, and benefits.

The conceptual visualization of the metaverse's technological ecosystem in healthcare, depicted in Figure 10, employs the metaphor of an umbrella. Technology is depicted as an interconnected structure that serves as a foundation for immersive and interactive healthcare environments. The rain symbolizes the challenges that these technologies must successfully overcome to achieve their objectives. When technologies are implemented thoughtfully, acknowledging these challenges, key application domains flourish, and benefits arise. The metaphor is active; the healthcare professional who holds the umbrella emphasizes human agency and the role of users in adopting technology and navigating complexity. At the foundation of the figure, advantages such as personalized care, remote accessibility, and inclusive learning environments are illustrated as sprouting plants, symbolizing growth potential when technologies and their applications are implemented diligently. To ensure safe and effective adoption, healthcare organizations should prioritize usability, accessibility, and clinical validation. Training programs that prepare professionals for immersive environments while safeguarding privacy and data security are essential. Robust governance frameworks and interoperability standards are essential for addressing ethical, legal, and equity concerns. Aligning these efforts with the WHO's strategic objectives^43^ will help create a sustainable ecosystem that fosters innovation without compromising trust, inclusivity, or patient safety.

Integrating metaverse technology into healthcare and healthcare learning environments introduces cultural and social challenges that extend beyond technical skills acquisition.^121^ Successful adoption depends on innovation and understanding end users’ needs. Metaverse solutions should prioritize usability, comfort, and accessibility. Research should involve healthcare professionals, educators, and patients to ensure practical relevance. A participatory approach will improve trust, adoption, and alignment with real-world workflows and values. As metaverse technologies become more embedded in healthcare, research should also examine the cognitive and emotional dimensions of professional adaptation. Concepts such as meta-work^122^ offer perspectives on how individuals engage with immersive technologies, manage complexity, and sustain meaningful work. To realize the metaverse's full potential in healthcare, future research must move beyond conceptual mapping and conduct empirical studies that assess real-world effectiveness, usability, and equity. This includes clinical trials, comparative evaluations, and investigations into user experiences. Until more evidence accumulates, the metaverse should be viewed as a promising but still maturing approach that requires careful monitoring and evaluation.

### Study limitations

This mapping review shares similarities with scoping reviews and evidence gap maps^52^ but emphasizes thematic mapping over intervention assessment. It synthesizes literature reviews rather than primary studies to offer a second-order perspective on how the metaverse has been conceptualized. Computational methods support structural mapping but limit deep qualitative interpretations. Therefore, a scoping review, as described by Campbell et al.,^52^ might have offered richer insights into the practical applications of metaverse technologies in healthcare by allowing for a more detailed examination of study contexts, interventions, and outcomes. For instance, a more detailed mapping of the risk landscape is a suitable topic for future research, such as a scoping review. Moreover, instead of reviewing review articles, future studies should synthesize primary empirical evidence on metaverse applications in healthcare to assess real-world effectiveness.

The review included 49 articles, likely due to terminological ambiguity, which may have led to the underrepresentation of certain application domains, particularly those described using alternative terminology not retrieved with our keyword-based search strategies. Although our review consistently used terms for immersive technologies, such as extended reality (VR, AR, MR), holography, and haptic devices and intelligent systems, including AI, ML, IoT, DTs, wearable biosensors, cloud computing, and robotics, to describe the metaverse ecosystem, terminology across the literature remains inconsistent. Many included reviews discussed these technologies individually rather than conceptualizing the metaverse as an integrated ecosystem. Such a lack of standardization contributes to terminological ambiguity and may affect the comprehensiveness of literature searches. Although the term “metaverse” serves as a useful umbrella concept that captures the convergence of immersive technologies, many relevant studies may not have used it. The reliance on open-access databases and a single-screening process may have excluded relevant studies, and inconsistencies across databases limited comprehensiveness. The reliance on English-language sources also excluded non-English perspectives. Typically, mapping reviews concentrate on descriptive and numerical accounts of large evidence sets, frequently encompassing more than 80 studies.^52^ Most studies were published in 2024–2025, reflecting emerging trends rather than long-term impacts. The included publications varied in methodological quality and disciplinary focus, which may affect the comparability and generalizability of the mapping. As a mapping review, this study did not assess the risk of bias or intervention effectiveness. The hybrid approach added depth, but interpretation remains subjective and may introduce bias. AI-assisted validation supported consistency but could not replace human judgment. However, the convergence of findings from multiple analytical approaches resulted in a coherent and consistent thematic structure, strengthening the credibility of the results. Future studies should expand database coverage, refine keyword strategies, and incorporate double-screening protocols to achieve a broader, more representative dataset. This would help overcome current limitations related to terminological ambiguity and database scope.

## Conclusion

The metaverse is a rapidly evolving technological ecosystem with the potential to transform healthcare delivery and education. Our findings revealed three thematic clusters: (1) immersive therapeutic and educational applications, (2) immersive technologies for surgical training and simulation, and (3) integrated immersive and intelligent technologies for personalized and networked healthcare. These clusters illustrate a shift from conceptual exploration toward applied and system-level integration. The developed umbrella metaphor underscores that technological innovation alone is insufficient. Success depends on governance, strategy implementation, and human-centered design. Hence, aligning the developments with the WHO's strategic objectives of collaboration, implementation, governance, and human-centered systems is essential to ensure ethical, inclusive, and evidence-based adoption. To realize the potential of the metaverse, stakeholders must prioritize usability, accessibility, and clinical validation, supported by robust ethical and regulatory frameworks.

Most of the metaverse evidence remains preliminary, and challenges such as high costs, technical limitations, ethical concerns, and unequal access persist. Research and development must generate robust evidence on clinical and educational outcomes, address governance and ethical challenges, ensure inclusivity, foster collaboration, and adhere to WHO objectives. Until these conditions are met, the metaverse should be regarded as a promising but immature approach requiring careful monitoring and policy alignment.

## Supplemental Material

sj-docx-1-dhj-10.1177_20552076261431602 - Supplemental material for Immersive futures in healthcare: A mapping review of review articles on the metaverse Supplemental material, sj-docx-1-dhj-10.1177_20552076261431602 for Immersive futures in healthcare: A mapping review of review articles on the metaverse by Pauliina Rikala, Minna Ylönen, Mads Solberg, Charlott Sellberg, Ville Heilala, Teuvo Antikainen, Miguel Munoz, Tommi Kärkkäinen and Raija Hämäläinen in DIGITAL HEALTH

sj-xlsx-2-dhj-10.1177_20552076261431602 - Supplemental material for Immersive futures in healthcare: A mapping review of review articles on the metaverse Supplemental material, sj-xlsx-2-dhj-10.1177_20552076261431602 for Immersive futures in healthcare: A mapping review of review articles on the metaverse by Pauliina Rikala, Minna Ylönen, Mads Solberg, Charlott Sellberg, Ville Heilala, Teuvo Antikainen, Miguel Munoz, Tommi Kärkkäinen and Raija Hämäläinen in DIGITAL HEALTH

sj-xlsx-3-dhj-10.1177_20552076261431602 - Supplemental material for Immersive futures in healthcare: A mapping review of review articles on the metaverse Supplemental material, sj-xlsx-3-dhj-10.1177_20552076261431602 for Immersive futures in healthcare: A mapping review of review articles on the metaverse by Pauliina Rikala, Minna Ylönen, Mads Solberg, Charlott Sellberg, Ville Heilala, Teuvo Antikainen, Miguel Munoz, Tommi Kärkkäinen and Raija Hämäläinen in DIGITAL HEALTH

sj-docx-4-dhj-10.1177_20552076261431602 - Supplemental material for Immersive futures in healthcare: A mapping review of review articles on the metaverse Supplemental material, sj-docx-4-dhj-10.1177_20552076261431602 for Immersive futures in healthcare: A mapping review of review articles on the metaverse by Pauliina Rikala, Minna Ylönen, Mads Solberg, Charlott Sellberg, Ville Heilala, Teuvo Antikainen, Miguel Munoz, Tommi Kärkkäinen and Raija Hämäläinen in DIGITAL HEALTH

sj-docx-5-dhj-10.1177_20552076261431602 - Supplemental material for Immersive futures in healthcare: A mapping review of review articles on the metaverse Supplemental material, sj-docx-5-dhj-10.1177_20552076261431602 for Immersive futures in healthcare: A mapping review of review articles on the metaverse by Pauliina Rikala, Minna Ylönen, Mads Solberg, Charlott Sellberg, Ville Heilala, Teuvo Antikainen, Miguel Munoz, Tommi Kärkkäinen and Raija Hämäläinen in DIGITAL HEALTH

sj-py-6-dhj-10.1177_20552076261431602 - Supplemental material for Immersive futures in healthcare: A mapping review of review articles on the metaverse Supplemental material, sj-py-6-dhj-10.1177_20552076261431602 for Immersive futures in healthcare: A mapping review of review articles on the metaverse by Pauliina Rikala, Minna Ylönen, Mads Solberg, Charlott Sellberg, Ville Heilala, Teuvo Antikainen, Miguel Munoz, Tommi Kärkkäinen and Raija Hämäläinen in DIGITAL HEALTH

sj-py-7-dhj-10.1177_20552076261431602 - Supplemental material for Immersive futures in healthcare: A mapping review of review articles on the metaverse Supplemental material, sj-py-7-dhj-10.1177_20552076261431602 for Immersive futures in healthcare: A mapping review of review articles on the metaverse by Pauliina Rikala, Minna Ylönen, Mads Solberg, Charlott Sellberg, Ville Heilala, Teuvo Antikainen, Miguel Munoz, Tommi Kärkkäinen and Raija Hämäläinen in DIGITAL HEALTH

sj-py-8-dhj-10.1177_20552076261431602 - Supplemental material for Immersive futures in healthcare: A mapping review of review articles on the metaverse Supplemental material, sj-py-8-dhj-10.1177_20552076261431602 for Immersive futures in healthcare: A mapping review of review articles on the metaverse by Pauliina Rikala, Minna Ylönen, Mads Solberg, Charlott Sellberg, Ville Heilala, Teuvo Antikainen, Miguel Munoz, Tommi Kärkkäinen and Raija Hämäläinen in DIGITAL HEALTH
