# Supplementary material for: Immersive futures in healthcare: A mapping review of review articles on the metaverse
Source: Digit Health. 2026 Mar 13;12:20552076261431602. doi: 10.1177/20552076261431602 (PMC12988307; doi:10.1177/20552076261431602)
Supplement: sj-docx-4-dhj-10.1177_20552076261431602 - Supplemental material for Immersive futures in healthcare: A mapping review of review articles on the metaverse [file sj-docx-4-dhj-10.1177_20552076261431602.docx]

# APPENDIX 4 Preprocessing and topic modeling details (Immersive Futures in Healthcare: A Mapping Review of Review Articles on the Metaverse)

**APPENDIX 4 Table 1.** Custom stopword list

| **Stopwords** | | | |
| --- | --- | --- | --- |
| abstract | access | addition | additionally |
| address | addressing | adoption | allow |
| allowing | allows | among | analyse |
| analysis | another | application | applied |
| apply | approach | are | area |
| areas | article | articles | aspect |
| aspects | assessment | associated | author |
| authors | available | background | based |
| become | better | beyond | bibliographic |
| bibliometric | can | case | challenges |
| characteristics | chart | choosing | citation |
| citations | cluster | clusters | compared |
| concerns | conclude | conducted | considered |
| content | context | could | countries |
| country | create | criteria | critical |
| crucial | current | data | database |
| decision | demographics | design | develop |
| development | diagram | different | diverse |
| document | documents | download | driven |
| due | early | effect | effectively |
| effects | efficacy | efficient | eligibility |
| enable | enables | enabling | enhance |
| enhanced | enhances | ensure | ensuring |
| evaluation | even | example | exclusion |
| existing | exluded | facilitate | factor |
| factors | field | fig | figure |
| findings | flow | focus | following |
| form | found | fully | furthermore |
| future | group | has | have |
| help | high | highlights | identify |
| ieee | image | impact | important |
| improve | improved | improving | include |
| included | includes | including | increased |
| industry | information | insights | instance |
| integrate | integrated | interest | introduction |
| is | issue | issues | journal |
| journals | key | keywords | knowledge |
| lead | leading | level | levels |
| like | limited | list | literature |
| low | made | main | major |
| make | making | many | may |
| method | methods | might | model |
| moreover | must | narrative | necessary |
| need | new | non | novel |
| number | objectives | offer | offering |
| offers | often | one | outcomes |
| overall | paper | papers | particularly |
| people | perform | population | possible |
| potential | present | presented | primary |
| prisma | procedure | procedures | process |
| progress | promising | proposed | provide |
| providing | publication | publications | published |
| quality | question | questions | range |
| real | reality | recarding | recent |
| record | records | reduce | reference |
| related | relevant | requires | research |
| researchers | result | results | review |
| revolutionize | sample | science | scientific |
| search | searches | section | see |
| select | selecting | selection | should |
| shown | shows | significant | size |
| specific | still | strategy | studies |
| study | summary | survey | synthesis |
| systematic | table | technology | term |
| terms | title | titles | topic |
| topics | total | trends | type |
| understanding | unique | use | used |
| user | users | using | utilized |
| various | via | view | virtual |
| volume | was | way | ways |
| well | were | will | within |
| word | world | year | years |

**APPENDIX 4 Table 2.** Preprocessing steps and parameters

| **Preprocessing steps and parameters** | | |
| --- | --- | --- |
| MALLET Command | mallet import-dir | --input metaversedata2 --output testi.mallet --keep-sequence --encoding UTF-8 --remove-stopwords --extra-stopwords stopwords.txt |
| MALLET Command | mallet train-topics (8 topics) | --input testi.mallet --num-topics 8 --num-iterations 1000 --optimize-interval 10 --output-state topic-state.gz --output-topic-keys topic-keys.txt --output-doc-topics doc-topics.txt |
| MALLET Command | mallet train-topics (6 topics) | --input testi.mallet --num-topics 6 --num-iterations 1000 --optimize-interval 10 --output-state topic-state.gz --output-topic-keys topic-keys.txt --output-doc-topics doc-topics.txt |
| LDA Parameter | Topics tested | 6 and 8 |
| LDA Parameter | Iterations | 1,000 |
| LDA Parameter | Alpha (α) | Optimized automatically every 10th iteration |
| LDA Parameter | Beta (β) | 0.01 (default) |
| LDA Parameter | Random seed | Not specified (MALLET default) |
| LDA Parameter | Stopwords | MALLET default + custom list (Appendix 2, Table 1) |
| LDA Parameter | Preprocessing | PDF → plain text, no stemming/lemmatization |
