# Supplementary material for: Immersive futures in healthcare: A mapping review of review articles on the metaverse
Source: Digit Health. 2026 Mar 13;12:20552076261431602. doi: 10.1177/20552076261431602 (PMC12988307; doi:10.1177/20552076261431602)
Supplement: sj-docx-5-dhj-10.1177_20552076261431602 - Supplemental material for Immersive futures in healthcare: A mapping review of review articles on the metaverse [file sj-docx-5-dhj-10.1177_20552076261431602.docx]

# APPENDIX 5 Statistical validation (Immersive Futures in Healthcare: A Mapping Review of Review Articles on the Metaverse)

**APPENDIX 5 Table 1. Large jumps in Ward’s agglomeration coefficients**

| **Stage** | **Coefficient** | **Δ from previous** | **% change** | **Interpretation** |
| --- | --- | --- | --- | --- |
| 37 | 0.928 | +0.110 | 13.4% | 13 clusters |
| 38 | 1.045 | +0.117 | 12.6% | 12 clusters |
| 39 | 1.185 | +0.140 | 13.4% | 11 clusters |
| 40 | 1.341 | +0.156 | 13.2% | 10 clusters |
| 41 | 1.538 | +0.197 | 14.7% | 9 clusters |
| 42 | 1.742 | +0.204 | 13.3% | 8 clusters |
| 43 | 2.115 | +0.373 | 21.4% | 7 clusters |
| 44 | 3.014 | +0.899 | 42.5% | 6 clusters |
| 45 | 4.060 | +1.046 | 34.7% | 5 clusters |
| 46 | 5.313 | +1.253 | 30.8% | CUT: k=3 retained |
| 47 | 6.903 | +1.590 | 29.9% | 2 clusters |
| 48 | 9.292 | +2.389 | 34.6% | 1 cluster (all merged) |

Notes. Ward’s agglomeration coefficient equals the increase in within-cluster variance at each merge. Large late-stage jumps (Stages 43–48) suggest cutting just before the jumps.

**APPENDIX 5 Table 2. Full agglomeration schedule**

| **Stage** | **Coefficient** | **Δ** | **% change** |
| --- | --- | --- | --- |
| 1 | 0.001 | — | — |
| 2 | 0.003 | +0.002 | 200.0% |
| 3 | 0.006 | +0.003 | 100.0% |
| 4 | 0.009 | +0.003 | 50.0% |
| 5 | 0.013 | +0.004 | 44.4% |
| 6 | 0.018 | +0.005 | 38.5% |
| 7 | 0.026 | +0.008 | 44.4% |
| 8 | 0.036 | +0.010 | 38.5% |
| 9 | 0.045 | +0.009 | 25.0% |
| 10 | 0.055 | +0.010 | 22.2% |
| 11 | 0.065 | +0.010 | 18.2% |
| 12 | 0.075 | +0.010 | 15.4% |
| 13 | 0.086 | +0.011 | 14.7% |
| 14 | 0.098 | +0.012 | 14.0% |
| 15 | 0.112 | +0.014 | 14.3% |
| 16 | 0.127 | +0.015 | 13.4% |
| 17 | 0.142 | +0.015 | 11.8% |
| 18 | 0.157 | +0.015 | 10.6% |
| 19 | 0.173 | +0.016 | 10.2% |
| 20 | 0.190 | +0.017 | 9.8% |
| 21 | 0.209 | +0.019 | 10.0% |
| 22 | 0.230 | +0.021 | 10.0% |
| 23 | 0.256 | +0.026 | 11.3% |
| 24 | 0.281 | +0.025 | 9.8% |
| 25 | 0.307 | +0.026 | 9.3% |
| 26 | 0.333 | +0.026 | 8.5% |
| 27 | 0.363 | +0.030 | 9.0% |
| 28 | 0.394 | +0.031 | 8.5% |
| 29 | 0.433 | +0.039 | 9.9% |
| 30 | 0.472 | +0.039 | 9.0% |
| 31 | 0.513 | +0.041 | 8.7% |
| 32 | 0.556 | +0.043 | 8.4% |
| 33 | 0.609 | +0.053 | 9.5% |
| 34 | 0.673 | +0.064 | 10.5% |
| 35 | 0.738 | +0.065 | 9.7% |
| 36 | 0.818 | +0.080 | 10.8% |
| 37 | 0.928 | +0.110 | 13.4% |
| 38 | 1.045 | +0.117 | 12.6% |
| 39 | 1.185 | +0.140 | 13.4% |
| 40 | 1.341 | +0.156 | 13.2% |
| 41 | 1.538 | +0.197 | 14.7% |
| 42 | 1.742 | +0.204 | 13.3% |
| 43 | 2.115 | +0.373 | 21.4% |
| 44 | 3.014 | +0.899 | 42.5% |
| 45 | 4.060 | +1.046 | 34.7% |
| 46 | 5.313 | +1.253 | 30.9% |
| 47 | 6.903 | +1.590 | 29.9% |
| 48 | 9.292 | +2.389 | 34.6% |

**APPENDIX 5 Table 3. One-way ANOVA and Welch robust test across clusters for topic probabilities (Ward solution)**

| **Topic** | **F (ANOVA)** | **p** | **Between SS** | **Total SS** | **η²** | **η² 95% CI** | **Levene’s p** | **Significant Tukey HSD pairs** | **Welch W** | **Welch df2** | **Welch p** |
| --- | --- | --- | --- | --- | --- | --- | --- | --- | --- | --- | --- |
| Topic 1 | 127.093 | <0.001 | 1.915 | 2.261 | 0.847 | [0.749, 0.888] | 0.002 | 2 > 1; 2 > 3 | 66.092 | 23.863 | <0.001 |
| Topic 2 | 8.054 | 0.001 | 0.347 | 1.339 | 0.259 | [0.054, 0.425] | 0.003 | 1 > 2; 1 > 3 | 6.883 | 29.822 | 0.003 |
| Topic 3 | 12.204 | <0.001 | 0.650 | 1.875 | 0.347 | [0.119, 0.503] | <0.001 | 3 > 1; 3 > 2 | 8.891 | 22.502 | 0.001 |
| Topic 4 | 14.996 | <0.001 | 0.598 | 1.515 | 0.395 | [0.161, 0.543] | <0.001 | 3 > 1; 3 > 2 | 12.068 | 28.625 | <0.001 |
| Topic 5 | 7.282 | 0.002 | 0.308 | 1.281 | 0.240 | [0.042, 0.407] | <0.001 | 1 > 2; 1 > 3 | 6.351 | 29.813 | 0.005 |
| Topic 6 | 4.300 | 0.019 | 0.161 | 1.021 | 0.158 | [0.003, 0.323] | <0.001 | 1 > 3 | 5.854 | 23.327 | 0.009 |

Notes. Ward clusters labeled 1–3. η² thresholds: small ≈0.01, medium ≈0.06, large ≈0.14. Levene’s p<0.05 indicates heteroscedasticity; when violated, Welch’s ANOVA provides robust inference. Post hoc: Tukey HSD (α=0.05) reported for descriptive completeness.

**APPENDIX 5 Table 4. Crosstab/Chi-square summary by topic vs. clusters**

| **Topic** | **Pearson χ² (df)** | **Asymptotic p** | **Likelihood ratio χ² p** | **Fisher–Freeman–Halton exact p (MC)** | **Linear by linear χ²** | **L×L Asymptotic p** | **L×L MC p (2 sided)** |
| --- | --- | --- | --- | --- | --- | --- | --- |
| Topic 1 | 98.000 (96) | 0.424 | 0.236 | 1.000 | 0.012 | 0.911 | 0.910 |
| Topic 2 | 98.000 (96) | 0.424 | 0.236 | 1.000 | 8.178 | 0.004 | 0.003 |
| Topic 3 | 98.000 (96) | 0.424 | 0.236 | 1.000 | 15.399 | <0.001 | <0.001 |
| Topic 4 | 98.000 (96) | 0.424 | 0.236 | 1.000 | 11.385 | <0.001 | <0.001 |
| Topic 5 | 98.000 (96) | 0.424 | 0.236 | 1.000 | 8.434 | 0.004 | <0.001 |
| Topic 6 | 98.000 (96) | 0.424 | 0.236 | 1.000 | 7.479 | 0.006 | 0.003 |

Notes: MC = Monte Carlo exact significance (10,000 samples; 99% CI). Due to many expected counts < 5, Monte Carlo estimates are preferred over asymptotic p-values. N of valid cases per table = 49.
